# CD96 marks a phenotypically distinct checkpoint-associated HCV-specific CD8+ T-cell subset featuring memory-associated states

**DOI:** 10.3389/fimmu.2026.1883007

**Published:** 2026-07-13

**Authors:** Maximilian Knapp, Christin Ackermann, Melanie Wittner, Claudia Beisel, Leon Cords, Tim Westphal, Liz Lam, Silke Kummer, Robin Woost, Sven Peine, Sven Pischke, Christoph Schultheiß, Ansgar W. Lohse, Andreas Walker, Jörg Timm, Mascha Binder, Julian Schulze zur Wiesch

**Affiliations:** 1 Infectious Diseases Unit, I. Department of Medicine, University Medical Center Hamburg-Eppendorf, Hamburg, Germany; 2 University of Cologne, Faculty of Medicine and University Hospital Cologne, Clinic III for Internal Medicine, Cologne, Germany; 3 Institute of Hematopathology Hamburg HpH, Hamburg, Germany; 4 German Center for Infection Research (DZIF), Partner Site Hamburg-Lübeck-Borstel-Riems, Hamburg, Germany; 5 PROVIREX Genome Editing Therapies GmbH, Hamburg, Germany; 6 Department of Internal Medicine IV & Division of Infectious Disease and Tropical Medicine, University Hospital Heidelberg, Heidelberg, Germany; 7 German Center for Infection Research (DZIF), Partner Site Heidelberg, Heidelberg, Germany; 8 Department of Oncology, Hematology and Bone Marrow Transplantation with Division of Pneumology, University Medical Center Eppendorf, Hamburg, Germany; 9 Institute of Transfusion Medicine, University Medical Center Hamburg-Eppendorf, Hamburg, Germany; 10 Division of Medical Oncology, University Hospital Basel, Basel, Switzerland; 11 Laboratory of Translational Immuno-Oncology, Department of Biomedicine, University and University Hospital Basel, Basel, Switzerland; 12 European Reference Network for Hepatological Diseases (ERN RARE-LIVER), Hamburg, Germany; 13 Institute of Virology, Medical Faculty, University Hospital Düsseldorf, Heinrich-Heine-Universität, Düsseldorf, Germany

**Keywords:** CD8+ T cells, CD96 (TACTILE), hepatitis C virus (HCV), immune checkpoint, T-cell exhaustion

## Abstract

**Introduction:**

Hepatitis C virus (HCV) infection frequently progresses to chronicity, where persistent antigen exposure drives differentiation of virus-specific CD8+ T cells toward an exhausted phenotype. This state is defined by progressive upregulation of inhibitory receptors and functional impairment of antigen-specific T cells. Because patients span distinct clinical stages from acute infection to spontaneous resolution or therapy induced sustained virological response, HCV provides a unique model to study how T-cell exhaustion is established, maintained, and potentially reversed. While PD1 and TIGIT are well established in this context, CD96 (TACTILE) is a less-characterized receptor that shares its ligand CD155 with TIGIT and DNAM-1 and may modulate CD8+ T-cell differentiation and function.

**Methods:**

To evaluate the role of CD96 across infection stages, we used HCV-specific MHC class I tetramers combined with high-dimensional flow cytometry to phenotypically profile virus-specific CD8+ T-cells *ex vivo* in 32 patients spanning acute, subacute, chronic, spontaneously resolved infection, and post-treatment sustained virological response. We assessed CD96 co-expression with PD1/TIGIT, transcription factors (TCF1, TOX, IRF4, T-bet, NR2F6), and relevant differentiation and exhaustion markers.

**Results:**

CD96+ HCV-specific CD8+ T cells consistently increased relative to bulk across all stages of HCV infection, with approximately four-fold higher frequencies than bulk CD96+ CD8+ T cells (p<0.0001), and highest frequencies of 51,13% observed in the chronic group. Because CD96 expression was predominantly distributed continuously rather than strictly bimodal, we also report CD96 MFI as a continuous measurement alongside CD96+ frequencies. Phenotypically, HCV-specific and bulk CD96+ CD8+ T cells were enriched for IRF4+, TOX+ and TCF1+ subsets, suggesting possible reduced effector features and increased memory-associated characteristics. Notably, the CD96+ PD1+ TIGIT+ subset was about five times higher in HCV-specific than in bulk CD8+ T-cell populations (p<0.0001). In cross-sectional comparison, frequencies of CD96+ PD1+ TIGIT+ HCV-specific CD8+ T cells decreased, while the CD96- PD1- TIGIT- counterparts expanded.

**Discussion:**

These findings identify CD96 as a marker of a phenotypically distinct checkpoint-associated HCV-specific CD8+ T-cell subset featuring memory-associated rather than terminally exhausted states. Whether this phenotypic association has functional or therapeutic significance for combination immune therapy will require future studies, including direct functional assays of sorted CD96+ versus CD96- HCV-specific CD8+ T cells.

## Graphical Abstract

**Figure:**
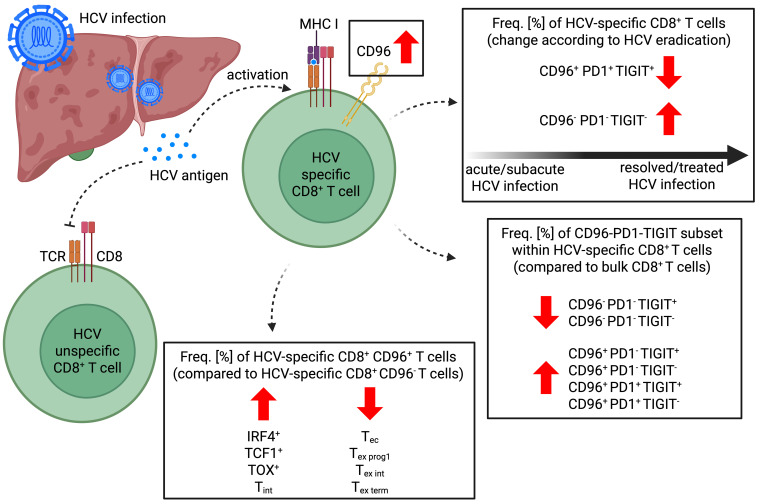
HCV-specific CD8+ T cells show higher frequencies of CD96+ cells compared to bulk CD8+ T cells. Within the HCV-specific CD8+ T cells, CD96+ cells show higher frequencies of IRF4+, TCF1+, TOX+ and Tint cells compared to the CD96- subset, whereas the frequencies of Tec, Tex prog1, Tex int and Tex term are decreased in the CD96+ subset. Comparing the co-expression of CD96, PD1 and TIGIT reveals that HCV-specific CD8+ T cells demonstrate decreased frequencies of CD96+ PD1- TIGIT+ and CD96- PD1- TIGIT- subsets, but increased frequencies of CD96+ PD1- TIGIT+, CD96+ PD1- TIGIT-, CD96+ PD1+ TIGIT+ and CD96+ PD1+ TIGIT- subsets compared to bulk CD8+ T cells. According to HCV eradication during disease evolution CD96+ PD1+ TIGIT+ HCV-specific CD8+ T cells decrease, whereas the CD96- PD1- TIGIT- subset increases. Created in BioRender. Knapp, M. (2026) https://BioRender.com/cvpjafk.

## 1 Introduction

With approximately 50 million affected people and 1 million new infections each year, hepatitis C virus (HCV) infection remains a global health burden with relevant health and economic effects (1, 2). 30% of patients spontaneously clear the infection, while 70% of patients progress to chronicity (1, 3). Today, direct-acting antivirals (DAAs) cure over 90% of cases (4–6). Due to its well-defined and stage-dependent clinical course, encompassing acute, chronic, and resolved phases as well as treatment-induced viral clearance, HCV infection provides a valuable model to study T-cell exhaustion driven by persistent antigen exposure (7, 8). This framework enables for the analysis of dynamic changes in T-cell function and phenotype across different infection stages and after viral eradication.

Exhausted T cells represent a phenotype with diminished immune functions. These cells exhibit impaired function due to upregulation of immune-checkpoint molecules that send negative signals, thereby restricting T-cell activation and effector responses (9–11). The discovery of “programmed cell death protein 1” (PD1) (12–15) and “cytotoxic T-lymphocyte-associated protein 4” (CTLA4) (15–17) marks a milestone in translational immunology. Subsequently, treatment with checkpoint-blocking antibodies has transformed cancer therapy (18–20). “T cell activation, increased late expression” (TACTILE, CD96) represents an emerging immune checkpoint with unique properties compared to well-studied inhibitory receptors. First described in 1992 as a novel surface antigen on various immune cells (21), mucosal-associated invariant (MAIT) and CD8+ T cells exhibit the highest CD96 expression among T-cell subsets, while γδ T cells and regulatory T cells (T_regs_) display lower levels (22). Comparing different effector subsets of CD4+ and CD8+ T cells, CD96 expression is highest in memory CD4+ and CD8+ T cells, especially enriched in Th1 CD4+ cells with a proinflammatory phenotype (23, 24).

The CD96 molecule binds to its ligands “nectin-like protein 5” (NECL-5, CD155) (25–27) and “poliovirus receptor-related 1” (PVRL-1, CD111) (27, 28). Unlike other well-established checkpoints, CD96 competes with the co-stimulatory receptor “DNAX accessory molecule-1” (DNAM-1) (29) and the co-inhibitory receptor “T-cell immune receptor with Ig and ITIM domains” (TIGIT) (30). Both of which also bind CD155 (25, 31–35), positioning it as a critical regulatory node that may shift the balance between T-cell activation and inhibition. Multiple tumor models demonstrate enhanced tumor control, increased IFNγ production, or improved response to anti-PD1 therapy by blocking or genetically deleting CD96 (36–43). Dual TIGIT/CD96 blockade using bispecific antibodies like BMS-986442 (AGEN1777) achieves superior tumor control compared to single-agent TIGIT blockade (44) suggesting that CD96 is a candidate component of combination checkpoint strategies.

Despite emerging relevance in chronic viral infection and tumour immunity, CD96 remains substantially less well characterized on human virus-specific CD8+ T cells than PD1 or TIGIT, providing a clear rationale for its phenotypic characterisation in the well-defined human HCV setting. This study examined CD96 expression on HCV-specific CD8+ T cells at all stages of HCV infection, assessing its co-expression with PD1 and TIGIT along with key markers of activation and differentiation. The goal was to provide a detailed phenotypic, rather than functional or mechanistic, characterization of the CD96+ HCV-specific CD8+ T-cell subset. Given the low frequency of HCV-specific CD8+ T cells often below conventional detection limits, we conducted comprehensive MHC class I tetramer-based *ex vivo* phenotyping (45) which can detect virus-specific CD8+ T cells at extremely low frequencies (46, 47) to identify distinct CD96-associated cellular states. We hypothesized that CD96 is enriched on HCV-specific CD8+ T and characterizes a specific subset of antigen-specific T cells with a unique phenotypic profile co-expressed with inhibitory receptors.

## 2 Material and methods

### 2.1 Ethic statement

Written informed consent was given by all study participants. The study was performed in accordance with the declaration of Helsinki and was reviewed by the local ethics board of the Ärztekammer Hamburg (WF14-09, PV4780, PV4081).

### 2.2 Patient cohort

Cryopreserved peripheral blood mononuclear cells (PBMC) from patients with confirmed hepatitis C virus infection at various disease stages were used for HCV-specific MHC class I tetramer staining and enrichment. All samples were collected at the Infectious Diseases Unit of the University Medical Center Hamburg-Eppendorf. Infection status was classified into acute (aHCV, n=7), subacute (sHCV, n=4), chronic (cHCV, n=12), spontaneously resolved (rHCV, n=7) and post treatment (tHCV, n=8) based on clinical records, serology, liver enzymes, symptoms and estimated infection duration derived from peripheral blood mononuclear cells (PBMC) Chronic HCV infection was defined as failure to clear the virus six months after infection (1, 48). Cases lacking definitive clinical or immunological features of acute or chronic infection were classified as subacute (HCV RNA positive, > 12 weeks post-infection but < 6 months). These cases reflect intermediate clinical reality, in which not all infections can be definitively classified as acute or chronic. Patients post-treatment (tHCV) achieved a sustained virologic response (SVR), defined as the persistent absence of detectable HCV RNA in plasma ≥ 12 weeks after completion of antiviral therapy or, for patients with a shorter follow-up, at the last available timepoint post-therapy. **Supplementary Table 1** provides demographic, clinical and virologic information for each HCV-infected patient.

### 2.3 HLA typing

High-definition molecular HLA class I and II typing by polymerase chain reaction specific sequence oligonucleotide (PCR-SSO) using the kit SSO LabType (One Lambda, Canoga Park, CA, USA) was done at the Institute of Transfusion Medicine at the University Medical Center Hamburg-Eppendorf based on the manufacturer’s instructions.

### 2.4 Sequencing of HCV isolates

Sequencing procedure of patient isolates was done as previously described at the Institute of Virology of the University Hospital Düsseldorf (49–51). Briefly, according to the manufacturer’s instruction the QIAamp Viral RNA Kit (Qiagen, Hilden, Germany) was used to extract RNA. Reverse transcription was carried out using SuperScript III (Invitrogen™, Thermo Fisher, Germany) and the reverse primer Oligo d(A) (52). A two-step nested PCR using GoTaq Polymerase (Promega, Walldorf, Germany) and genotype-specific primers were used to generate HCV amplicons. PCR conditions consisted of an initial denaturation step at 94 °C for 120 s, followed by 35 cycles of 30 s at 94 °C, 30 s at 55 °C, and 160 s at 72 °C, with a final extension at 72 °C for 10 min. Geneious version 10.2.6 (Biomatters, Auckland, New Zealand) was then used to directly evaluate PCR products.

### 2.5 MHC class I tetramer staining and enrichment

MHC class I tetramer-associated magnetic bead enrichment was conducted as previously described (30, 45, 49). The used HCV-specific MHC class I tetramers with HLA types and corresponding amino acid sequences can be found in **Table 1**. In brief, cryopreserved PBMC were thawed up and incubated with PE-labeled HLA class I tetramers matching the patients` HLA type. The enrichment of the MHC class I tetramer-positive HCV-specific cells was performed using anti-PE microbeads with LS columns for Magnetic Activated Cell Sorting (MACS) technology (Miltenyi Biotec, Bergisch Gladbach, Germany) according to the manufacturer’s protocol. The pre-enriched, depleted, and enriched fractions were used for surface and intracellular staining and for multicolor flow cytometry. Representative tetramer enrichment and total number of HCV-specific CD8+ T cells of each patient is shown in **Supplementary Figure 1**; **Supplementary Table 3**. The frequencies of HCV-specific MHC class I tetramer+ CD8+ T cells were calculated as previously described (53).

**Table 1:** Used tetramers for identification of HCV-specific CD8+ T cells.

HLA-A molecule	Target protein	Amino acid position	Sequence
HLA-A*01:01	NS3	1436 - 1444	ATDALMTGY
HLA-A*02:01	NS3	1406 - 1415	KLVALGINAV
HLA-A*02:01	NS3	1073 - 1081	CINGVCWTV
HLA-A*24:02	E2	717 - 725	EYVLLLFLL

The table shows the HLA-A molecules used for our tetramer stainings with corresponding target proteins, amino acid position and sequence of the epitopes.

### 2.6 Multicolor flow cytometry

PBMC were used for multiparametric flow cytometry after MHC class I tetramer staining and enrichment. To exclude death cells from the following analysis, LIVE/DEAD™ Fixable Near-IR dye (Invitrogen™, Thermo Fisher, Germany) was used as specified by the manufacturer. Surface staining was performed using an indirect staining with purified anti-CD352 (clone: W19035D, catalog-number: 332302, BioLegend) and BUV563 anti-ratIgG1/IgG2 (clone: G28-5, catalog-number: 748690, BD), as well as with fluorochrome-conjugated surface antibodies, including BUV805 anti-CD8 (clone: SK1, catalog-number: 612889, BD), BUV737 anti-CD73 (clone: AD2, catalog-number: 612812, BD), BUV661 anti-PD1 (clone: EH12.1, catalog-number: 750260, BD), BUV615 anti-CD38 (clone: HIT2, catalog-number: 751138, BD), BUV496 anti-CD28 (clone: CD28.2, catalog-number: 741168, BD), BUV395 anti-CD62L (clone: SK11, catalog-number: 565219, BD), BV785 anti-CD127 (clone: A019D5, catalog-number: 351330, BioLegend), BV650 anti-CD4 (clone: RPA-T4, catalog-number: 300536, BioLegend), BV605 anti-TIGIT (clone: A15153G, catalog-number: 372712, BioLegend), BV510 anti-CD39 (clone: A1, catalog-number: 328220, BioLegend), PerCP-Cy5.5 anti-CD69 (clone: FN50, catalog-number: 310926, BioLegend), PE-Dazzle anti-CD96 (clone: NK92.39, catalog-number: 338414, BioLegend), APC-Cy7 anti-CD14 (clone: 63D3, catalog-number: 367108, BioLegend), APC-Cy7 anti-CD19 (clone: HIB19, catalog-number: 302236, BioLegend), and AF700 anti-CD3 (clone: UCHT1, catalog-number: 300424, BioLegend). After surface staining, cells were fixed and permeabilized using the eBioscience™ Foxp3/transcription factor staining buffer set (Invitrogen™, Thermo Fisher, Germany). For intracellular staining, BV711 anti-T-bet (clone: 4B10, catalog-number: 644819, BioLegend), BV421 anti-TCF1 (clone: S33-966, catalog-number: 566692, BD), FITC anti-NR2F6 (polyclonal, catalog-number: orb222499, biorbyt), PE-Cy7 anti-IRF4 (clone: 3E4, catalog-number: 25-9858-82, Thermo Fisher) and APC anti-TOX (clone: REA473, catalog-number: 130-118-335, Miltenyi Biotec) fluorochrome-conjugated antibodies were used. The flow cytometry panel is provided in **Supplementary Table 2**. Measurements were performed using a BD FACSymphony™ A3 machine and FACSDiva version 8 for Windows (BD Bioscience, San Jose, USA). Because CD96 expression on human CD8+ T cells was predominantly continuously distributed rather than strictly bimodal, CD96 was analyzed both as a frequency and as a continuous variable (mean fluorescence intensity, MFI). Samples were included in subset-level analyses only if at least 20 tetramer+ HCV-specific CD8+ T-cell events were recorded after enrichment. Representative enrichment and per-sample tetramer+ event counts are shown in **Supplementary Figure 1**; **Supplementary Table 3**.

### 2.7 Statistical analysis

FlowJo™ version 10.9.0 software (BD Bioscience, San Jose, USA) was used for the analysis of all flow cytometric data. The basic gating strategy for identification of CD8+ HCV-specific T cells can be found in **Supplementary Figure 2**. Statistical analysis was performed using GraphPad Prism version 10 (GraphPad software, San Diego, CA). For comparison of individual non-paired samples, the Mann-Whitney test was used. Paired analyses were performed using Wilcoxon matched-pairs signed rank test. Results with a p-value smaller than 0.05 were considered significant, where *, **, ***, and **** indicate p-values between 0.01 and 0.05, 0.001 and 0.01, 0.0001 and 0.001, and < 0.0001, respectively. Box-plots or individual values with or without mean and standard deviation were used for data expression. All frequencies mentioned in the text have been mathematically rounded to two decimal places. The exact values were used for the statistical analysis.

## 3 Results

### 3.1 Study cohort, demographics and clinical characteristics

The study cohort included 32 clinically characterized patients with hepatitis C virus infection (**Supplementary Table 1**), categorized by HCV infection status: acute (aHCV, n=7), subacute (sHCV, n=4), chronic (cHCV, n=12) and spontaneously resolved HCV infection (rHCV, n=7), as well as patients post treatment (tHCV, n=8). HCV-specific CD8+ T cells were analyzed and phenotyped via HCV-specific HLA class I tetramers and 20-parameter flow cytometry as previously described (30, 45, 49). To capture dynamics of the immunological status, six patients were assessed at two distinct stages of infection (T_1_ and T_2_, aHCV or sHCV and rHCV or tHCV). Except for the post-treatment group, all patients were treatment-naive at the time of sample collection. Detailed demographic and clinical characteristics for all patients are available in **Table 2**; **Supplementary Table 1**. The summarized demographic and clinical data for each patient group based on clinically documented infection status are presented in **Table 3**. After establishing this cohort, we next aimed to characterize the expression patterns of CD96 on HCV-specific CD8+ T cells across these clinical stages.

**Table 2:** Short demographics and clinical characteristics from all patients of the study cohort.

Patient	Status	HLA-A	Age	Sex	AST [U/L]	ALT [U/L]	Viral load [IU/mL]	Genotype	Therapy
HCV01	aHCV	*02:01, *03:01	51	♀	n/a	n/a	n/a	1a	–
HCV02	rHCV	*02:01, *11:01	49	♂	12	19	<	n/a	–
HCV03	tHCV	*02:01, -	48	♂	8	18	<	1a	IIb study IFN vs. IFN + Ribavirin
HCV04	cHCV	*02:01, *03:01	41	♂	42	48	n/a	3a	IFN + Ribavirin
HCV05	cHCV	*24:02, *31:01	31	♀	47	40	400,000	3a	–
HCV06	sHCV tHCV	*02:01, -	32 33	♂	159 56	364 56	n/a <	n/a	IFN
HCV07	sHCV	*01:01, *30:01	29	♂	39	58	40,000,000	1a	Ribavirin + NS3/4A-I + nnRNAP-I
HCV08	cHCV	*01:01, *33:01	47	♂	n/a	n/a	3,000,000	3a	–
HCV09	cHCV	*02:01, -	65	♂	36	36	20,000,000	2b	IFN + Ribavirin
HCV10	aHCV rHCV	*01:01, *23:01	54	♀	485 11	1126 11	30,000,000 <	3a	–
HCV11	rHCV	*01:01, *24:02	47	♀	34	35	<	n/a	–
HCV12	sHCV tHCV	*02:01, *11:01	22 23	♀	167 13	265 8	400,000 <	1a	IFN + Ribavirin
HCV13	rHCV	*02:01, *03:01	50	♂	n/a	n/a	<	n/a	–
HCV14	aHCV tHCV	*02:01, *32:01	36 38	♂	22 20	36 34	300,000 <	1a	IFN + Ribavirin
HCV15	rHCV	*02:01, *68:01	61	♂	23	26	<	n/a	–
HCV16	tHCV	*03:01, *24:02	44	♂	21	19	<	1b	Ombitasvir + Paritaprevir + Ritonavir + Dasabuvir
HCV17	sHCV	*01:01, *02:01	33	♀	87	212	41,200	1a	Sofosbuvir + Ledispavir
HCV18	cHCV	*02:01, *11:01	55	♂	88	179	757,000	1a	Sofosbuvir + Ledispavir
HCV19	aHCV rHCV	*01:01, *03:01	52 53	♂	558 29	957 58	41,400 <	3	–
HCV20	tHCV	*02:01, *03:01	42	♂	17	27	<	1a	Glecaprevir + Pibrentasvir
HCV 21	rHCV	*01:01, *02:01	48	♀	20	20	<	n/a	–
HCV22	aHCV tHCV	*02:01, *24:02	42	♂	900 18	1,488 19	11,900,000 <	3	Sofosbuvir + Ledispavir
HCV23	tHCV	*01:01, *02:01	60	♂	n/a	n/a	n/a	1a	Sofosbuvir + Ledispavir
HCV24	cHCV	*01:01, -	77	♀	69	74	272,000	1a	Glecaprevir + Pibrentasvir
HCV25	cHCV	*02:01, *03:01	49	♂	22	61	1,860,000	4	Grazoprevir
HCV26	cHCV	*02:01, *29:02	69	♂	61	70	1,060,000	3	–
HCV27	cHCV	*02:01, *03:01	51	♂	24	40	13,000,000	1a	–
HCV28	aHCV	*02:01, *24:02	30	♂	690	2155	4,590,000	3	–
HCV29	cHCV	*24:02, *25:01	20	♂	62	202	552,000	3	–
HCV30	cHCV	*02:01, *03:01	72	♀	39	53	920,000	1b	–
HCV31	cHCV	*01:01, *02:01	46	♂	90	126	1,480,000	3	Glecaprevir + Pibrentasvir
HCV32	aHCV	*02:01, *31:01	40	♂	85	465	128,000	1b	Glecaprevir + Pibrentasvir

The table shows patient ID, infection state, HLA-A, age at sample collection, sex, aspartate aminotransferase (AST), alanine aminotransferase (ALT), viral load, HCV genotype and therapy. All laboratory parameters are measured at date of sample collection.Bold values indicate the patients HLA types matching the used tetramers.

**Table 3:** Summarized demographic and clinical data for each group based on clinically documented infection status.

Status	n	Sex	Age [years]	Viral load [IU/mL]	Albumin [g/L]	Bilirubin [mg/dL]	AST [U/L]	ALT [U/L]	GGT [U/L]	AP [U/L]	CRP [mg/L]	Quick [INR]
aHCV	7	♀ 2 ♂ 5	44 [36 - 54]	7,826,567 [41,400 - 30,000,000] n/a n=1	38,1 [43 - 34,6] n/a n=1	2,15 [0,7 - 6,4] n/a n=1	457 [22 - 900] n/a n=1	1034 [36 - 2155] n/a n=1	645 [261 - 1396] n/a n=1	160 [60 - 318] n/a n=2	12,5 [11 - 14] <5 n=3 n/a n=2	0,99 [0,97 - 1] n/a n=4
sHCV	4	♀ 2 ♂ 2	29 [22 - 33]	13,480,400 [41,200 - 40,000,000] n/a n=1	41,5 [39,4 - 43] n/a n=1	0,5 [0,4 - 0,7] n/a n=0	113 [39 - 167] n/a n=0	225 [58 - 364] n/a n=0	93 [29 - 209] n/a n=0	60 [52 - 74] n/a n=0	<5 n=1 n/a n=3	0,96 [0,9 - 1,05] n/a n=1
cHCV	12	♀ 3 ♂ 9	52 [20 - 77]	3,936,455 [40,000 - 20,000,000] n/a n=1	40,8 [34,3 - 47,2] n/a n=3	0,56 [0,4 - 0,9] n/a n=3	53 [22 - 90] n/a n=1	85 [36 - 202] n/a n=1	122 [21 - 645] n/a n=1	82 [47 - 167] n/a n=1	<5 n=8 n/a n=4	1,13 [1 - 1,3] n/a n=9
rHCV	7	♀ 4 ♂ 3	52 [47 - 61]	viral load of all samples is below detection limit	41,6 [39 - 44] n/a n=3	0,38 [0,2 - 0,5] n/a n=3	22 [11 - 34] n/a n=1	28 [11 - 58] n/a n=1	52 [16 - 173] n/a n=1	80 [59 - 139] n/a n=1	<5 n=1 n/a n=6	1,02 [1 - 1,04] n/a n=5
tHCV	8	♀ 1 ♂ 7	41 [23 - 60]	viral load is below detection limit n=7 n/a n=1	42 [40 - 47] n/a n=2	0,55 [0,4 - 0,7] n/a n=6	22 [8 - 56] n/a n=1	26 [8 - 56] n/a n=1	39 [19 - 94] n/a n=2	44 [36 - 61] n/a n=3	8 n=1 <5 n=4 n/a n=3	1,02 [0,94 - 1,14] n/a n=5
all	38	♀ 12 ♂ 26	46 [20 - 77]	6,535,080 [41,200 - 40,000,000] below detection limit n=14 n/a n= 4	41 [40 - 47,2] n/a n=10	0,9 [0,2 - 6,4] n/a n=13	119 [8 - 900] n/a n=4	247 [8 - 2155] n/a n=4	186 [16 - 1396] n/a n=5	85 [36 - 318] n/a n=7	11 [8 - 14] <5 n=17 n/a n=18	1,02 [0,9 - 1,3] n/a n=24

The table shows sex, age, viral load, albumin, bilirubin, aspartate aminotransferase (AST), alanine aminotransferase (ALT), gamma-glutamyl transferase (GGT), alkaline phosphatase (AP), C-reactive protein (CRP) and international normalized ratio (INR) for the different HCV subgroups. The values are calculated as arithmetic mean. If values were not available (mentioned as n/a), arithmetic mean was calculated out of the known samples.

### 3.2 HCV-specific CD8+ T cells show elevated CD96 expression across all stages of disease with highest frequency and MFI in chronic infection

**Figure 1:**
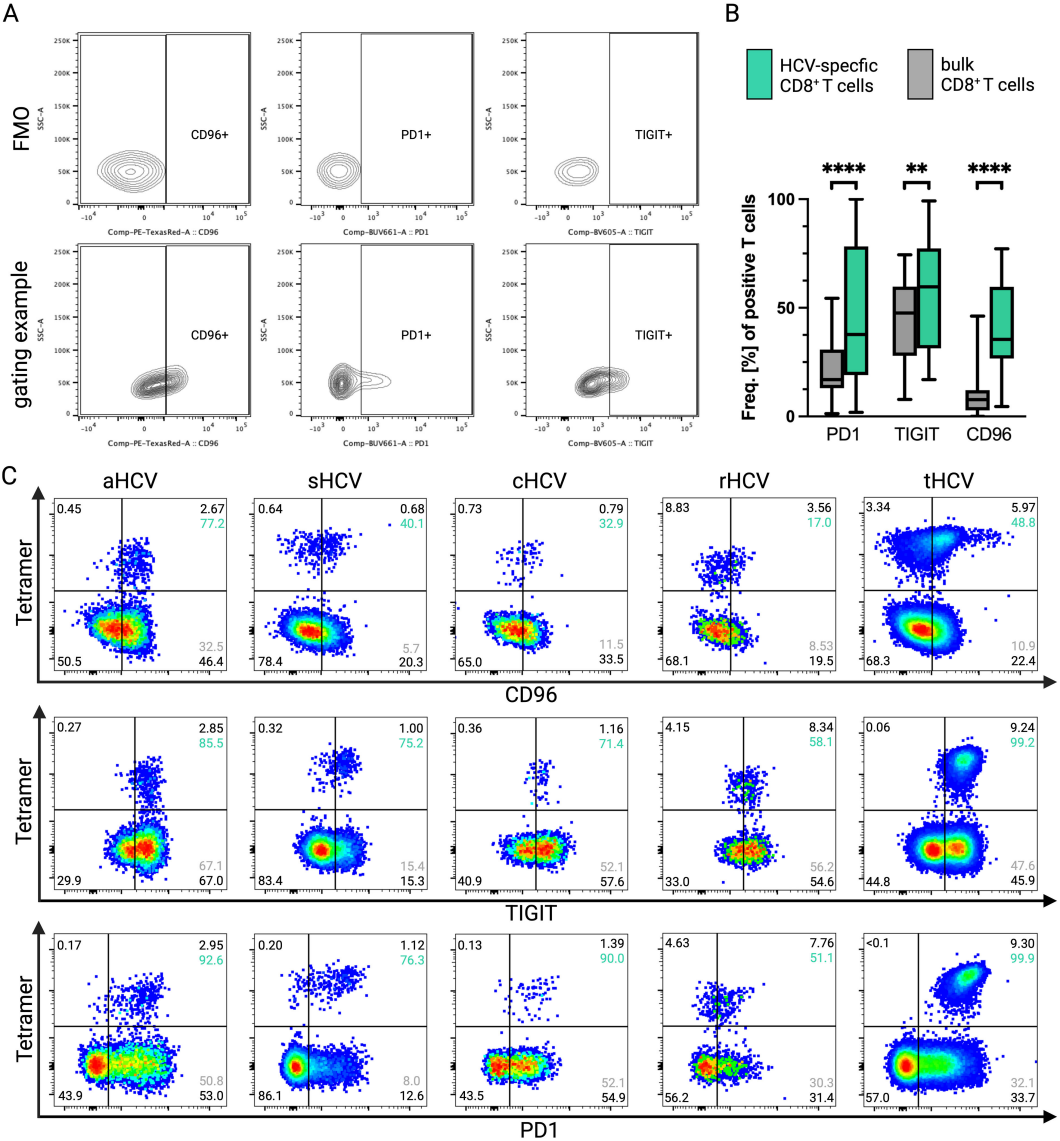
**(A)** Gating definition for CD96+, PD1+ and TIGIT+ cells based on FMOs with exemplary gates for each marker. **(B)** Frequencies of PD1+, TIGIT+ and CD96+ cells gated on bulk (grey) vs. HCV-specific (cyan) CD8+ T cells. **(C)** Representative plots for distribution of CD96+, TIGIT+ and PD1+ Tet+ and Tet- cells for one sample of each patient group gated on CD8+ T cells out of the enriched fraction (black). The frequencies shown in cyan represent the HCV-specific CD8+ T cells from the enriched fraction and the frequencies in grey represent the bulk CD8+ T cells out of the pre-enrichment fraction used for statistical analysis. Created in BioRender. Knapp, M. (2026) https://BioRender.com/6j4rflx. *, **, ***, and **** indicate p-values between 0.01 and 0.05, 0.001 and 0.01, 0.0001 and 0.001, and < 0.0001, respectively.

PD1 is a well-characterized immune checkpoint in chronic viral infections, including HCV, where its upregulation on virus-specific CD8+ T cells is associated with impaired effector function (14, 54–57). Furthermore, blocking the PD1 signaling pathway can restore CD8+ T-cell function in chronic HCV infection (13) and may also impact HCV viremia (58). Similarly, TIGIT, which shares the ligand of CD96 (25–27, 31–34), is also upregulated on HCV-specific T cells (30), and exerts inhibitory effects on immune function (59–61). Therefore, the aim was to compare the frequencies of PD1+, TIGIT+, and CD96+ cells (**Figure 1A**) in bulk and HCV-specific CD8+ T-cell subsets As expected, HCV-specific CD8+ T cells exhibited significantly higher frequencies of PD1+ (46,58% vs. 23,03%, p<0,0001) and TIGIT+ (57,09% vs. 45,13%, p=0,0014) cells compared to bulk CD8+ T cells. Notably, the frequencies of CD96+ cells were also significantly increased in the HCV-specific CD8+ T-cell subset compared to bulk (41,02% vs. 10,84%, p<0,0001) (**Figure 1B**).

**Figure 2:**
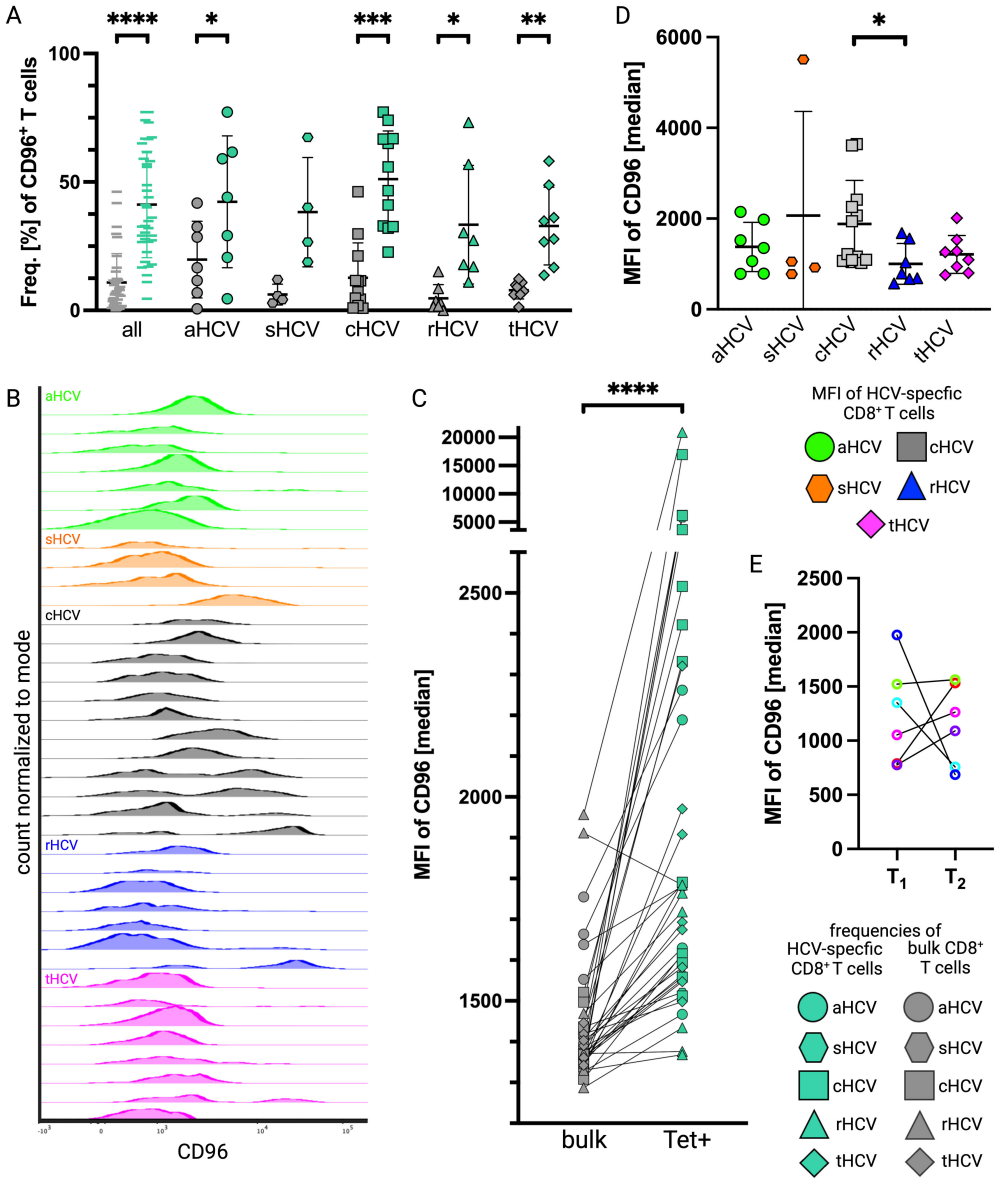
**(A)** Frequencies of CD96+ cells gated on bulk (grey) vs. HCV-specific (cyan) CD8+ T cells with and without differentiation between different stages of HCV infection. **(B)** Expression of CD96 vs. count as normalized to mode from patients with acute (n=7, green), subacute (n=4, orange), chronic (n=12, black), resolved (n=7, blue) and treated (n=8, pink) HCV infection on HCV-specific CD8+ T cells. **(C)** MFI of CD96 on bulk (grey) and HCV-specific (cyan) CD8+ T cells. Matched values of the same patient relate to the black lines. **(D)** MFI of CD96 of HCV-specific CD8+ T cells distinguished between the different stages of HCV infection. **(E)** MFI of CD96 of HCV-specific CD8+ T cells of six patients with follow-up samples at first (T_1_, aHCV or sHCV) and second timepoint (T_2_, rHCV or tHCV). Paired values of the patients are shown in same colours. Created in BioRender. Knapp, M. (2026) https://BioRender.com/pdgbiho. *, **, ***, and **** indicate p-values between 0.01 and 0.05, 0.001 and 0.01, 0.0001 and 0.001, and < 0.0001, respectively.

Next, the expression dynamics of CD96 across clinical stages was characterized. HCV-specific CD8+ T cells showed higher frequencies in acute (42,29% vs. 17,32%, p=0,0156), chronic (51,13% vs. 12,69%, p=0,0005), spontaneously resolved (33,37% vs. 4,74%, p=0,0156) and treated (32,84% vs. 7,90%, p=0,0078) infection compared to bulk CD8+ T cells, with peak levels observed in the chronic group (**Figure 2A**). Comparing the frequencies of CD96+ bulk and HCV-specific CD8+ T cells across the different disease stages revealed no significant differences.

Because CD96 expression was mostly continuously distributed rather than strict bimodal, especially in acute, subacute, resolved, and treated patients, CD96 was analyzed both by frequency and as MFI. This analysis (**Figure 2B**) showed that CD96 expression on CD96+ CD8+ T cells is significantly increased on HCV-specific compared to bulk cells (3626,47 vs. 1709,88, p<0,0001) (**Figure 2C**). Moreover, in the HCV-specific subset, the MFI showed a statistical trend toward increased levels in the chronic group compared to the other groups (**Figure 2D**), with a significantly higher MFI of CD96 in cHCV patients compared to the rHCV patients (1882,33 vs. 1001,29, p=0,0130). A comparative analysis of samples from patients collected during (sub-)acute stages and after spontaneous resolution or treatment did not show significant differences in CD96 MFI (MFI 1244,33 vs. 1149, p>0,9999) (**Figure 2E**). The MFI of PD1 significantly decreased after viral clearance (1667 vs. 541,83, p=0,0312) (**Supplementary Figure 3C**), and TIGIT showed a similar trend, although it was not statistically significant (2823 vs. 1732,83, p=0,0938) (**Supplementary Figure 4C**). No predictive factors for changes in CD96 expression after viral clearance were identified from patient characteristics (**Supplementary Table 1**).

### 3.3 CD96+ HCV-specific CD8+ T cells are marked by increased frequencies of IRF4+, TCF1+ and TOX+ cells

**Figure 3:**
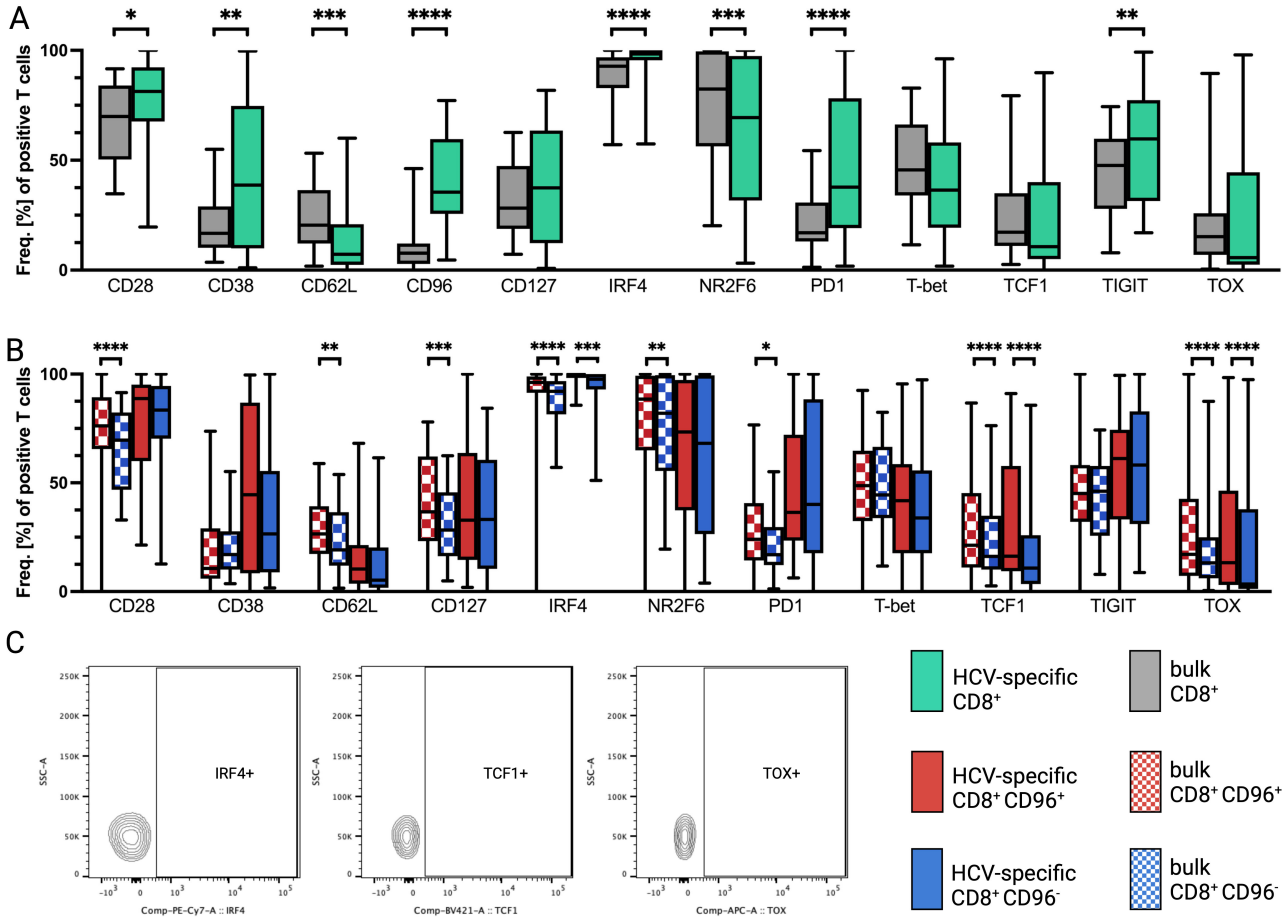
**(A)** Frequencies of target+ cells gated on bulk (grey) vs. HCV-specific (cyan) CD8+ T cells. **(B)** Frequencies of target+ cells gated on CD96+ bulk (red tiled) and HCV-specific (red) vs. CD96- bulk (blue tiled) and HCV-specific (blue) CD8+ T cells. **(C)** Gating definition for IRF4+, TCF1+ and TOX+ cells based on FMOs. Created in BioRender. Knapp, M. (2026) https://BioRender.com/e1zfzl7. *, **, ***, and **** indicate p-values between 0.01 and 0.05, 0.001 and 0.01, 0.0001 and 0.001, and < 0.0001, respectively.

Comparing a broad panel of proteins, including lineage markers (CD3, CD4, CD8, CD14, CD19), adenosine-signaling pathways (CD38, CD39, CD73), immune checkpoints (PD1, TIGIT, CD96), activation marker (CD28), transcription factors (IRF4, NR2F6, T-bet, TCF1, TOX), and markers of various functional T cells (CD62L, CD127, CD69, Slamf6), allowed the characterization of the expression signature of HCV-specific versus bulk CD8+ T cells. The HCV-specific subset showed significantly higher frequencies of CD28+ (75,89% vs. 66,90%, p=0,0341), CD38+ (41,17% vs. 20,79%, p=0,0015), IRF4+ (95,82% vs. 88,36%, p<0,0001), PD1+ (46,58% vs. 23,03%, p<0,0001) and TIGIT+ (57,09% vs. 45,13%, p=0,0014) cells, while the frequencies of CD62L+ (14,47% vs. 24,27%, p<0,0001) and NR2F6+ (61,30% vs. 76,72%, p=0,0003) cells were significantly decreased (**Figure 3A**). For CD39/CD73 subsets (**Supplementary Figure 5A**), the frequencies of CD39+ CD73+ (1,83% vs. 0,70%, p=0,0060) and CD39+ CD73- (19,52% vs. 4,95%, p=0,0006) cells were significantly higher in the HCV-specific CD8+ T-cell subset compared to the bulk population, while the frequencies of CD39- CD73- (52,17% vs. 59,73%, p=0,0253) cells were significantly lower (**Supplementary Figure 5B**).

Given that HCV-specific T cells were predominantly CD96+, we examined how these cells differed from the CD96- population. We found significantly higher frequencies of IRF4+ (98,39% vs. 94,35%, p=0,0002), TCF1+ (29,67% vs. 19,97%, p<0,0001) and TOX+ (27,23% vs. 20,80%, p<0,0001) cells in the CD96+ subset compared to the CD96- HCV-specific CD8+ T-cells (**Figure 3B**).

Analysis of the CD96+ bulk CD8+ T-cell subset revealed significantly elevated frequencies of CD28+ (74,78% vs. 65,76%, p<0,0001), CD62L+ (27,79% vs. 23,85%, p=0,0054), CD127+ (40,40% vs. 31,21%, p=0,0001), IRF4+ (89,59% vs. 88,07%, p<0,0001), NR2F6+ (78,49% vs. 75,98%, p=0,0022), PD1+ (27,55% vs. 21,82%, p=0,0109), TCF1+ (29,17% vs. 22,90%, p<0,0001) and TOX+ (27,85% vs. 20,31%, p<0,0001) cells in the CD96+ compared to the CD96- bulk CD8+ T-cell subset (**Figure 3B**). For the CD39/CD73 subsets, the CD96+ population tended to exhibit increased frequencies of the CD39+ CD73- subset compared to the CD96- population, while lower frequencies of the CD39- CD73+ and CD39- CD73- subsets were observed. However, these differences were statistically significant only for the overall CD8+ T cells with the CD39+ CD73- (9,99% vs. 3,65%, p=0,0006) and CD39- CD73- (54,42% vs. 59,93%, p=0,0236) subsets (**Supplementary Figure 5C**).

### 3.4 CD96+ HCV-specific CD8+ T cells show deviant frequencies of functional T-cell phenotypes compared to their CD96- counterparts

**Figure 4:**
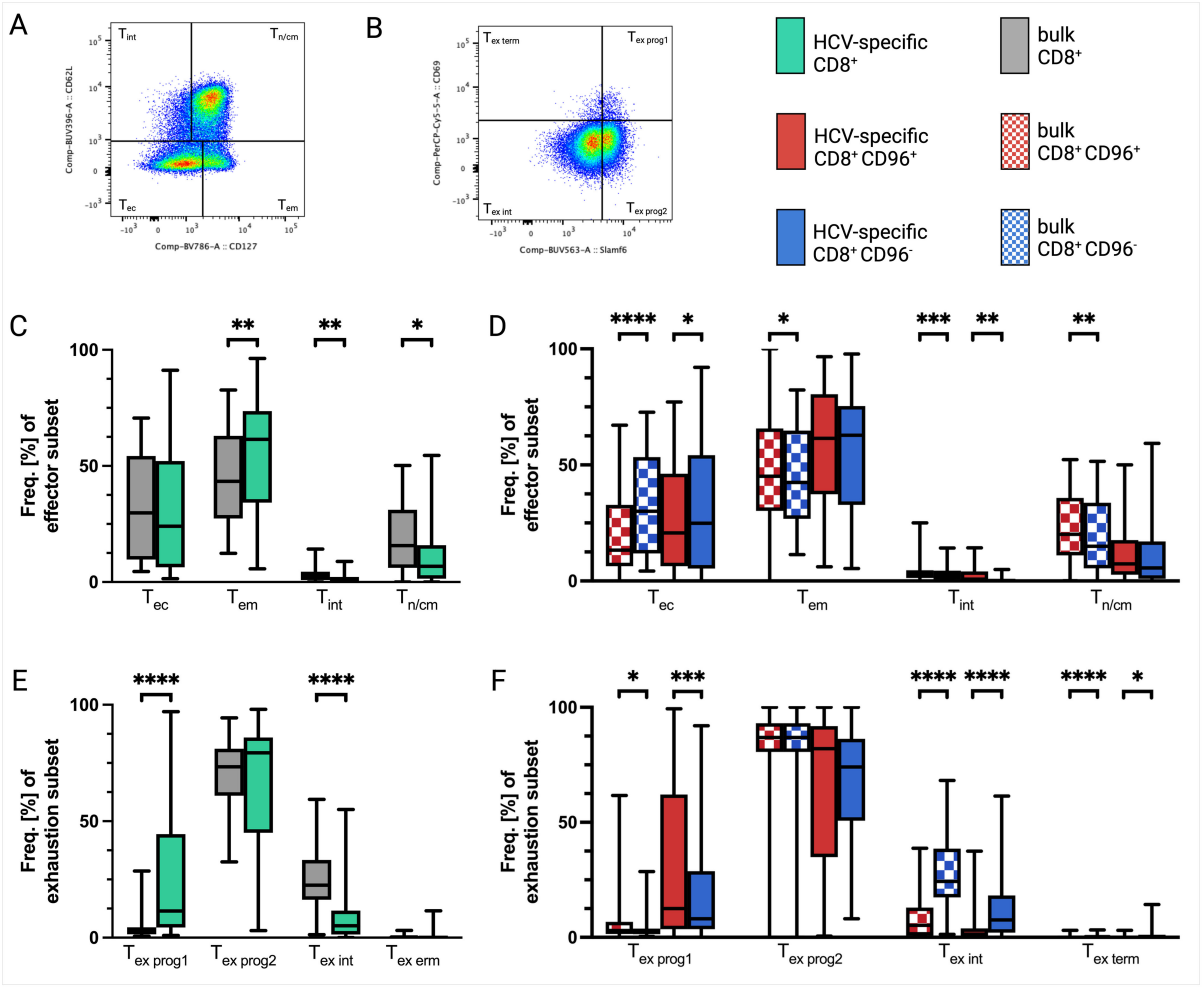
**(A)** Representative gating for identification of naïve/central memory (T_n/cm_, CD127+ CD62L+), effector (T_ec_, CD127- CD62L-), intermediate (T_ec_, CD127- CD62L+), and effector memory (T_em_, CD127+ CD62L-) CD8+ T cells. **(B)** Representative gating for identification of progenitor 1 (T_ex prog1_, Slamf6+ CD69+), progenitor 2 (T_ex prog2_, Slamf6+ CD69-), intermediate (T_ex int_, Slamf6- CD69-), and terminal exhausted (T_ex tern_, Slamf6- CD69+) CD8+ T cells. **(C)** Frequencies of effector subsets gated on bulk (grey) vs. HCV-specific (cyan) CD8+ T cells. **(D)** Frequencies of effector subsets gated on CD96+ bulk (red tiled) and HCV-specific (red) vs. CD96- bulk (blue tiled) and HCV-specific (blue) CD8+ T cells. **(E)** Frequencies of exhaustion subsets gated on bulk (grey) vs. HCV-specific (cyan) CD8+ T cells. **(F)** Frequencies of exhaustion subsets gated on CD96+ bulk (red tiled) and HCV-specific (red) vs. CD96- bulk (blue tiled) and HCV-specific (blue) CD8+ T cells. Created in BioRender. Knapp, M. (2026) https://BioRender.com/larw0y7. *, **, ***, and **** indicate p-values between 0.01 and 0.05, 0.001 and 0.01, 0.0001 and 0.001, and < 0.0001, respectively.

To evaluate the functional development of the CD8+ T cells, we classified cells based on CD127 and CD62L expression into effector (T_ec_, CD127- CD62L-), effector memory (T_em_, CD127+ CD62L-), intermediate (T_int_, CD127- CD62L+) and naïve/central memory (T_n/cm_, CD127+ CD62L+) CD8+ T cells (62, 63) (**Figure 4A**). This CD127/CD62L-based scheme functions as a simplified phenotypic framework rather than as a definitive lineage classification, and the T_n/cm_ designation reflects the fact that naïve and central memory cells cannot be reliably distinguished with the current marker set. HCV-specific CD8+ T cells exhibited significantly higher frequencies of T_em_ (56,02% vs. 44,91%, p=0,0038) but significantly lower frequencies of T_int_ (1,44% vs. 3,19%, p=0,0026) and T_n/cm_ (12,67% vs. 20,35%, p=0,0028) cells compared to bulk CD8+ T cells (**Figure 4C**).

A comparative analysis of CD96+ and CD96- cells showed that the CD96+ subset had significantly lower frequencies of T_ec_ in both HCV-specific (26,50% vs. 29,72%, p= 0,0243) and bulk (20,76% vs. 32,09%, p<0,0001) populations. Conversely, T_int_ frequencies were notably higher in HCV-specific (2,53% vs. 0,81%, p=0,0032) and bulk (4,48% vs. 3,10%, p=0,0006) subsets. For T_em_ (49,05% vs. 44,79%, p=0,0298) and T_n/cm_ (23,08% vs. 20,03%, p=0,0058), only the CD96+ bulk, and not the HCV-specific CD8+ T cells, showed significantly increased frequencies compared to CD96- cells, although similar trends were observed in the HCV-specific population (**Figure 4D**).

Finally, the interconnections between CD96 and exhaustion markers was investigated. While on average 41,02% of all HCV-specific CD8+ T cells were CD96+, the TIGIT+ HCV-specific CD8+ T cells showed increased frequencies of 58,86% CD96+ cells, and for the PD1+ HCV-specific CD8+ T-cell subset, 64,29% of cells were CD96 +. These increased frequencies of CD96+ cells were significant for all HCV-specific CD8+ T cells compared to the PD1+ (p< 0.0001) and the TIGIT+ (p< 0.0001) subset (data not shown). Using CD69 and Slamf6 to differentiate phenotypic subsets previously associated with distinct exhausted T cell subsets, the cells were categorized as quiescent resident progenitor 1 (T_ex prog1_, Slamf6+ CD69+), proliferative circulating progenitor 2 (T_ex prog2_, Slamf6+ CD69-), circulating mildly cytotoxic intermediate (T_ex int_, Slamf6- CD69-) and terminally exhausted resident (T_ex term_, Slamf6- CD69+) T cells (64) (**Figure 4B**). HCV-specific CD8+ T cells showed an increase in frequencies for T_ex prog1_ (25,38% vs. 3,74%, p<0,0001) but a decrease in T_ex int_ (9,40% vs. 24,78%, p<0,0001) compared to bulk (**Figure 4E**). For the T_ex prog1_ cells, significantly elevated frequencies were found in both the CD96+ bulk (6,37% vs. 3,53%, p=0,0129) and HCV-specific (31,01% vs. 19,66%, p=0,0005) CD8+ T-cell subsets compared to CD96- populations. The frequencies of the T_ex int_ (8,68% vs. 27,09%, p<0,0001 respectively 4,17% vs. 13,23%, p<0,0001) and T_ex term_ (0,34% vs. 0,56%, p<0,0001 respectively 0,28% vs. 1,18%, p=0,0175) subsets were significantly decreased in the CD96+ compared to the CD96- population for bulk as well as HCV-specific CD8+ T cells (**Figure 3F**). These SLAMF6/CD69 designations should be understood as phenotypic categories aligned with previously described exhaustion states, mainly because the underlying framework was based on murine chronic LCMV models and CD69 also indicates tissue-resident and recently activated cells (see Limitations).

### 3.5 CD96/PD1/TIGIT co-expression on HCV-specific CD8+ T cells significantly differs from the bulk CD8+ T-cell population and changes upon HCV eradication

PD1 and TIGIT are well-characterized inhibitory receptors linked to dysfunctional T cells. PD1 signaling reduces proliferation, cytokine production und cytolytic activity (65, 66) while TIGIT impairs IFNγ-production (67) and increases the suppressive function of T_regs_ (68). Both receptors are upregulated due to chronic antigen stimulation (30, 69). The CD96 and TIGIT signaling pathways are key regulatory components in T- and NK-cell modulation (70, 71). These receptors operate within an intricate network that includes molecules like DNAM-1, all sharing ligands to finely tune immune responses (27, 28, 31–34). Although CD96 (29, 36, 37, 72) and TIGIT (73–76) are predominantly known by their inhibitory roles, emerging evidence suggests that CD96 might also have co-stimulatory effects, especially in CD8+ T cells (77, 78). This functional duality positions CD96 and TIGIT as possible targets for immunotherapy, where strategic adjustment of their signaling pathways may potentiate immune responses (37, 44), making their co-expression in HCV infection a subject of particular interest.

**Figure 5:**
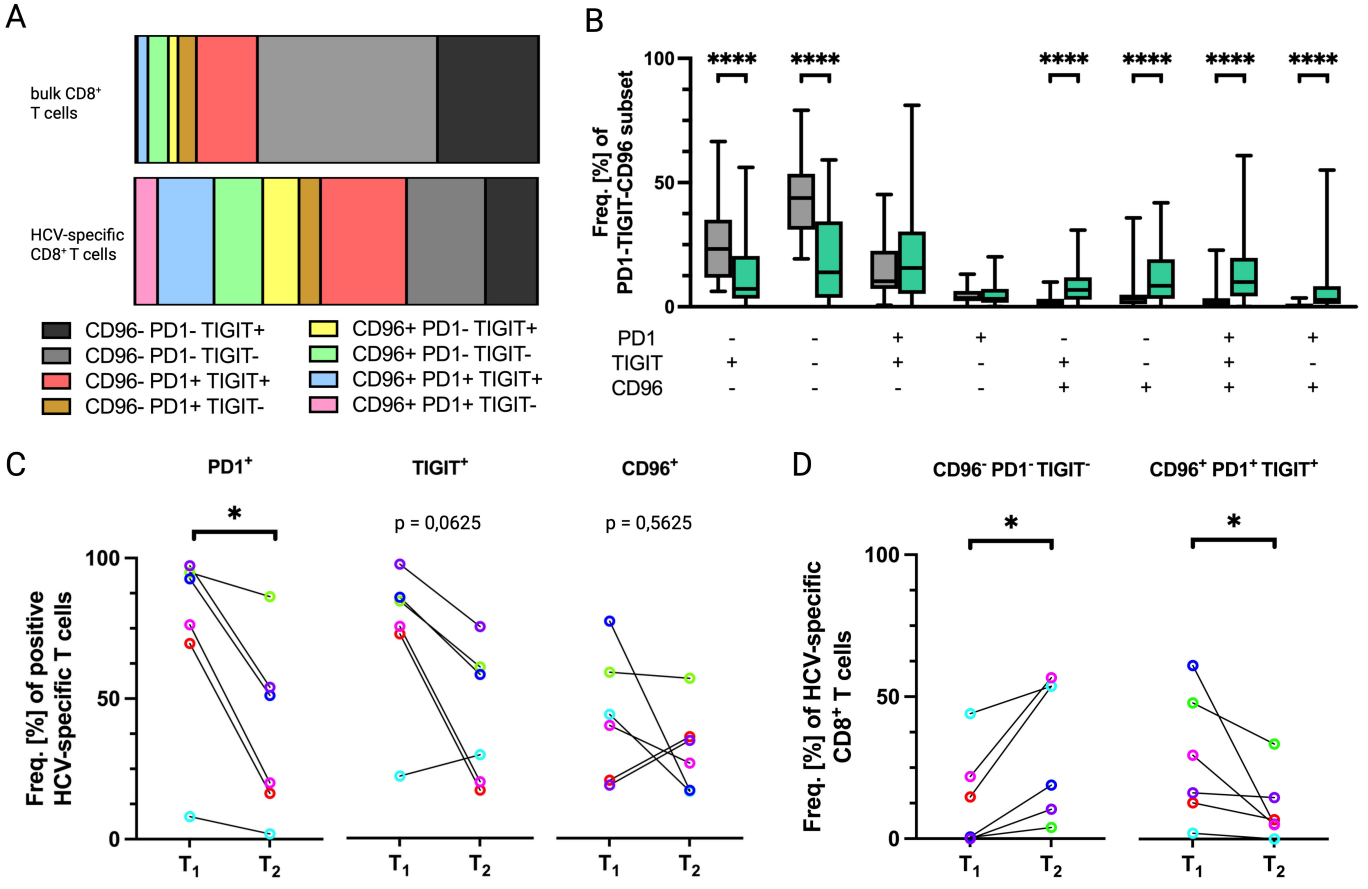
**(A)** Average frequencies as arithmetic mean of CD96-PD1-TIGIT subsets within bulk vs. HCV-specific CD8+ T cells. **(B)** Frequencies of CD96-PD1-TIGIT subsets gated on bulk (grey) vs. HCV-specific (cyan) CD8+ T cells. **(C)** Frequencies during course of HCV infection for PD1+, TIGIT+ and CD96+ HCV-specific CD8+ T cells of patients with follow-up samples at first (T_1_, aHCV or sHCV) and second timepoint (T_2_, rHCV or tHCV). **(D)** Frequencies during course of HCV infection for CD96- PD1- TIGIT- and CD96+ PD1+ TIGIT+ HCV-specific CD8+ T cells of patients with follow-up samples at first (T_1_, aHCV or sHCV) and second timepoint (T_2_, rHCV or tHCV). Created in BioRender. Knapp, M. (2026) https://BioRender.com/ei5w9am. *, **, ***, and **** indicate p-values between 0.01 and 0.05, 0.001 and 0.01, 0.0001 and 0.001, and < 0.0001, respectively.

To investigate this co-expression, we analyzed the average frequencies of CD96/PD1/TIGIT populations in bulk and HCV-specific CD8+ T-cell subset (**Figure 5A**). We observed lower frequencies of CD96- PD1- TIGIT+ and CD96- PD1- TIGIT- and higher frequencies of CD96+ PD1+ TIGIT+ and CD96+ PD1+ TIGIT- subsets in the HCV-specific CD8+ T-cell population. In-depth analysis revealed a significant decrease in the HCV-specific CD8+ T-cell population for the CD96- PD1- TIGIT+ (12,86% vs. 25,00%, p<0,0001) and CD96- PD1- TIGIT- (19,53% vs. 44,53%, p<0,0001) subsets, whereas the frequencies of the CD96+ PD1- TIGIT+ (8,98% vs. 2,39%, p<0,0001), CD96+ PD1- TIGIT- (12,05% vs. 5,04%, p<0,0001), CD96+ PD1+ TIGIT+ (13,98% vs. 2,65%, p<0,0001) and CD96+ PD1+ TIGIT- (6,00% vs. 0,76%, p<0,0001) subsets were significantly increased compared to bulk (**Figure 5B**).

Patients who spontaneously resolve HCV infection exhibit lower frequencies of PD1+ CD8+ HCV-specific T cells (13, 79), as well as patients after DAA therapy (80, 81). It has also been reported that frequency of TIGIT+ CD8+ HCV-specific T cells decreases following spontaneous resolution of infection (30). Finally, we assessed how HCV clearance influences the CD8+ T-cell phenotype using samples of six patients at two different time points of infection (T_1_ and T_2_, aHCV or sHCV and rHCV or tHCV). We observed a significant reduction of the PD1+ subset after HCV eradication (73,12% vs. 38,26%, p=0,0312) (**Figure 5C**). For the TIGIT+ cells, there was also a clear trend toward decrease after eradication, but it did not reach statistical significance (72,78% vs. 43,43%, p=0,0625). No clear trend clear trend was detected in the CD96+ subset, even when comparing clinical parameters (**Supplementary Table 1**). Regarding changes in CD96/PD1/TIGIT co-expression (**Supplementary Figure 6**), there was a significant increase in the CD96- PD1- TIGIT- subset (13,61% vs. 32,89%, p=0,0312), while the CD96+ PD1+ TIGIT+ subset significantly decreased (28,13% vs. 10,80%, p=0,0312) after HCV eradication (**Figure 5D**).

## 4 Discussion

This study presents the first comprehensive analysis of CD96 expression patterns across all stages of HCV infection. It offers a detailed, hypothesis-generating phenotypic characterization of CD96 expression on HCV-specific CD8+ T cells across all major clinical stages of HCV infection. HCV-specific CD8+ T cells consistently exhibit higher levels of CD96+ HCV-specific CD8+ T cells compared to overall bulk populations, showing distinct trends from the well-known checkpoints PD1 and TIGIT.

The increase in CD96+ frequencies among HCV-specific compared to bulk CD8+ T cells was significant across all HCV-infection stages except the subacute group, likely due to the small sample size (n=4). We kept the sHCV classification of patients to preserve clinical detail rather than merging groups to increase statistical power. This underscores that clinical HCV staging remains difficult, even at specialized centers. Despite lacking statistical significance, these subacute patients also showed a clear trend toward higher frequencies of CD96+ HCV-specific CD8+ T cells (**Figure 1C**). Overall, these findings implicate that CD96 upregulation may be an integral part of the anti-HCV immune response during ongoing viral antigen presentation. Since CD96 expression showed a primarily continuous distribution rather than a strictly bimodal pattern, both frequency-based and intensity-based analyses were considered. MFI analysis indicated that CD96 expression peaks during chronic infection. Although only statistically significant compared to the rHCV group – probably due to cohort size – our data suggest that CD96 expression is elevated on HCV-specific CD8+ T cells throughout all infection stages. This pattern sharply contrasts with the better-known profiles of PD1 and TIGIT (13, 30, 79).

HCV-specific CD8+ T cells displayed a distinct phenotypic profile compared to bulk populations, marked by higher frequencies of CD28+, CD38+, IRF4+, PD1+, and TIGIT+ cells, along with lower frequencies of CD62L+ and NR2F6+ cells. Notably, the CD96+ subset was consistently enriched with IRF4+, TCF1+, and TOX+ cells compared to the CD96- subset, indicating a specific transcriptional program which could be linked to a flexible, memory-like state rather than terminal exhaustion. While TOX is traditionally associated with exhaustion during chronic viral infections (49, 82, 83), it is also expressed in polyfunctional memory T cells, regardless of TCF1 co-expression (84). TCF1 has been connected to HCV-specific memory-like CD8+ T cells (85), self-renewal (86) and activated stem cell-like precursor central memory-like CD8+ T cells. It is also crucial for maintaining exhausted CD8+ T cells with stem-cell like phenotype (87). TCF1 expression is regulated by T-cell receptor signaling strength (88), and higher levels of CD8+ T cells expressing Transcription factor 7, which encodes for TCF1, are associated with improved responses to anti-PD1 therapy (89). The co-expression of TCF1 and TOX in CD96+ cells could indicate that these cells may display some degree of therapeutic plasticity, with a lower likelihood toward terminal exhaustion, than their CD96- counterparts. Additionally, IRF4 overexpression can promote T-cell exhaustion and downregulation of TCF1 (90). However, IRF4 also plays a key role in the differentiation, proliferation, and function of effector and memory cytotoxic CD8+ T cells. It regulates the production of interleukin-9 (IL-9) by IL-9-producing (Tc9) CD8+ T cells and the development of IL-17-producing (Tc17) CD8+ T cells (91). Overall, our findings may further support the idea that there is a link between CD96 expression and memory-like features of CD8+ T cells. Because we did not measure IFNγ, TNFα, IL-2, granzyme B, perforin, CD107a, proliferation upon peptide restimulation, or response to checkpoint blockade, the link between the observed CD96-associated IRF4/TCF1/TOX pattern and any specific functional state remains speculative. While our study mainly focused on phenotypic analysis, the presence of TCF1 in CD96+ HCV-specific T cells suggests functional relevance (89). TCF1+ PD1+ T cells represent progenitor-exhausted cells with stem cell-like properties that respond to immune-checkpoint therapy (92, 93). The maintenance of TCF1 expression in CD96+ cells, along with increased proportions of T_em_ (CD127+ CD62L-) and progenitor-exhausted (Slamf6+) characteristics (64), may indicates a higher probability for these cells to preserve proliferative and therapeutic potential with a less exhausted and non-viable phenotype. Additionally, the antigen-dependent, reversible regulation of the CD96+PD1+TIGIT+ triple-positive subset following viral clearance supports the idea that CD96 marks a more dynamic, non-terminally differentiated population. Consequently, our findings reinforce the connection between CD96 expression and memory-like CD8+ T-cell traits on a phenotypic based model, suggesting that this subset more likely maintains the proliferative and therapeutic capacities essential for effective immune-checkpoint blockade responses than the CD96- subset.

The CD127/CD62L-based classification served as a simplified phenotypic framework rather than a definitive delineation of human CD8+ T-cell differentiation states. HCV-specific CD8+ T cells were mainly CD127+ CD62L- effector memory (T_em_) cells with higher frequencies in the CD96+ subgroup compared to the CD96- group. This aligns with reports describing a polarization of HCV-specific CD8+ T cells toward memory-like traits, with molecular patterns of exhaustion remaining even after HCV clearance (94). Within the CD96+ subset, CD8+ CD127- CD62L- T_ec_ decreased, and CD127- CD62L+ T_int_ increased compared to CD96- cells. After activation, CD8+ T cells lower CD127 expression and then re-express it while reducing CD62L. Further differentiation depends on antigen exposure, leading to CD62L- CD127- T_ec_ or CD62L+ CD127+ T_cm_ if the antigen is absent. Upon stimulation, these T_cm_ cells produce strong IL-2 responses and proliferate but show limited immediate cytotoxicity. In contrast, T_ec_ cells exhibit high cytolytic activity but low proliferation. CD127+ CD62L- T_em_ display moderate effector function and recall capacity. Effector-type cells tend to protect against peripheral infections, whereas T_cm_ are more effective in systemic infections (63). This supports the idea that CD96+ CD8+ T cells are more memory-like. However, a more precise determination of CD8+ T-cell differentiation states would require additional markers such as CD45RA/CCR7, CD27/CD28, KLRG1, or CD57, which were not included in this panel. Future studies should ideally apply such extended panels and then analyze CD96 expression across differentiation states, rather than the other way round.

When using Slamf6 and CD69 to distinguish different states of exhausted T cells (64), the T_ex prog1_ subset is characterized by high TCF1 and CD28 expression, increased cytokine production, and strong proliferative capacity. T_ex prog2_ cells are highly proliferative, but produce fewer cytokines, have reduced TCF1 expression, and likely serve as a transitional stage toward T_ex int_. The T_ex int_ subset is a precursor to the dysfunctional T_ex term_, which loses T-bet and gains Eomes and TOX (64). CD69 can reduce proliferative capacity (95), but using it to define subsets has limitations. CD69 is associated with tissue-resident cells (96, 97), a population that may not be assessable using PBMC. It can also be assumed that the role of CD69 forming tissue-resident T cells depends on the specific tissue (98). Moreover, intrahepatic CD69+ CD8+ T cells show lower levels of cytotoxic proteins and T-bet (97). In mouse models, the CD69+ T_ex prog2_ and T_ex term_ cells are rare in peripheral blood but enriched in the spleen (64). Therefore, subset distributions in peripheral blood may differ significantly from those in various tissues. At different stages of HCV infection, CD8+ bulk and HCV-specific CD96+ T cells were mainly composed of Slamf6+ CD69- proliferative circulating T_ex prog2_ cells. CD96+ CD8+ T cells exhibited higher frequencies of T_ex prog1_ but lower frequencies of the T_ex int_ and T_ex term_ subsets compared to CD96- cells. In summary, this further supports the thesis that CD96+ cells may be linked to a slightly impaired functional state, yet still capable of change over time, and therefore are not definitively differentiated. However, we also recognize that these phenotypic classifications should be interpreted cautiously when applied to peripheral blood-derived HCV-specific CD8+ T cells. Therefore, the cells analyzed from blood may not accurately represent the liver compartment, and positivity for CD69 does not automatically indicates tissue residency or exhaustion. Phenotypes derived from PBMC may not fully capture intrahepatic T-cell states. The exhaustion trajectory model based on expression of CD69 and Slamf6 primarily originates from murine and chronic infection settings. To better understand these phenotypes and functional subsets, future studies should include tissue-resident T cells, especially from the liver as the primary site of HCV infection, where elevated antigen exposure may influence T-cell differentiation, function, and CD96 expression.

Because PD1, TIGIT, and CD96 may act as potential therapeutic targets for immune-checkpoint blockade and markers of exhaustion, their co-expression was analyzed across all stages of HCV infection. PD1 inhibits T-cell activation through PD-L1/PD-L2–mediated signaling that affects T-cell receptor signaling (99) and metabolic modulation (100). This leads to decreased T-cell function, including reduced proliferation and cytokine production (101). Normally, this helps maintain immune tolerance (102), but PD1 deficiency can cause immune overactivation and autoreactivity (103, 104). Tumor-associated PD-L1 upregulation suppresses anti-tumor responses, making the PD1 pathway a major therapeutic target (101). In chronic HCV-infection, intrahepatic CD8+ HCV-specific T cells show increased CTLA4, decreased CD28 and CD127, and fail to respond to anti-PD1 therapy, unlike their peripheral counterparts (105). TIGIT mediates intrinsic inhibitory signals in T and NK cells (73–76) through its immunoglobulin tail tyrosine (ITT)-like motif that binds “Growth Factor Receptor-Bound Protein 2” (Grb2), recruiting “Src homology region 2 domain-containing phosphatase-1” (SHP-1), and activating “phosphatidylinositol 3-kinase” (PI3K) and “mitogen-activated protein kinase” (MAPK) signaling (106). TIGIT also contains the “immunoreceptor tyrosine-based inhibition motif” (ITIM), which interacts with SHP-1, SHP-2, or the “Src homology 2 domain containing inositol polyphosphate 5-phosphatases 1 and 2” (SHIP1 or SHIP2) (107). Similarly, the YXXM motif of CD96 may bind the p85 subunit of PI3K via SH2, opens the possibility of inhibitory signaling potential (108). Therefore, it is conceivable that co-expression of PD1, TIGIT, and CD96 may result in a more inhibited, functionally restricted phenotype if their inhibitory signals are enhanced. However, because TIGIT and CD96 share the same ligand, there is also the possibility that co-expression could cause competitive binding, potentially limiting the function of one receptor. Considering the possibility of bidirectional signaling with co-inhibitory (36, 109) and co-stimulatory (23, 77) functions, these pathway connections may be highly relevant for further thesis generation and ongoing research. In our study, all CD96+ CD96/PD1/TIGIT subsets (CD96+ PD1+ TIGIT+, CD96+ PD1+ TIGIT-, CD96+ PD1- TIGIT+, CD96+ PD1- TIGIT-) were enriched in HCV-specific CD8+ T cells compared to the overall population, while CD96- PD1- TIGIT- and CD96- PD1- TIGIT+ CD8+ T cells declined. After HCV eradication, the frequencies of CD96+ PD1+ TIGIT+ cells decreased, whereas CD96- PD1- TIGIT- counterparts increased. This strengthens the assumption that ongoing antigen exposure sustains highly differentiated CD96+ PD1+ TIGIT+ T cells. It is consistent with the hypothesis that CD96 marks a dynamic, non-terminally differentiated population in chronic HCV infection, but it does not provide direct evidence for it.

The findings described above can carry translational therapeutic implications. AGEN1777 (TIGIT/CD96 bispecific antibody) shows improved anti-tumor response in preclinical models (44, 110). GSK is testing the anti-CD96 antibody GSK6097608 in patients with advanced solid tumors (111). Blocking of CD96 enhances CD8+ anti-tumor response in mouse models and CD96-deficient CD8+ T cells demonstrate greater inhibition of tumor growth than CD96+ cells (37). Preclinical studies indicate that CD96 signaling limits T-cell cytotoxicity (36), as depletion or blockade of CD96 boosts anti-tumor responses in multiple mouse models (38–41), especially when combined with anti-PD1 therapy. Higher CD96 expression on CD8+ T cells in cancer correlates with poorer responses to anti-PD1 therapy, and combining PD1 and CD96 blockade further improves CD8+ T-cell function (42, 43). In contrast, CD96^Hi^ CD4+ T cells exhibit a Th1/Th17 phenotype with high secretion of IFNγ (23) and a co-stimulatory effect of CD96 on CD8+ T cells has been observed (78). These findings support an inhibitory role for CD96 and strengthen the thesis that combined checkpoint blockade could be more effective than PD1 inhibition alone, although some co-stimulatory effects of CD96 may also be present. Nonetheless, the specific effects of CD96 blockade as a standalone treatment remain unclear and require further clinical research. Assuming that the indications described here are correct and CD96+ CD8+ T cells have more memory-like phenotypes with sustained proliferative capacity, this subset could be a target for checkpoint-based immunotherapy and warrants further functional investigation.

We also need to address the limitations of this study, primarily the moderate cohort size, which may have restricted the detection of additional significant differences. Cohort composition was limited by the MHC class I tetramer approach and the requirement for specific HLA types, potentially reducing generalizability, as different HLA types or epitopes can provoke distinct immune responses (112, 113). However, this approach provides the necessary resolution to characterize HCV-specific CD8+ T cells, which are often below the detection limits of conventional methods. Despite this variability, the consistent patterns observed in CD96 emphasize the patterns of this checkpoint signature. In this context, it should be acknowledged that the compared patient groups comprise relatively small sample sizes, particularly the subacute group. Nevertheless, we retained the current classification based on clinical documentation to ensure an unbiased representation of real-world clinical practice. We further have to acknowledge, that some observed differences between HCV-specific and bulk CD8+ T cells may reflect differences in activation and differentiation states, in addition to antigen specificity itself. Based on our findings for CD8+ HCV-specific T cells, we cannot draw broad conclusions about other infections. In this context, it has been reported that HIV-1 downregulates CD96 on CD4+ T cells as a viral escape (23). It is also important to note that our focus was on CD8+ T cells; however, the role of CD96 on CD4+ T cells also warrants investigation. Bunet et al. evaluated CD96 expression on CD8+ T cells in HIV infection and reported a reduction in HIV positive individuals, especially in typical progressors compared to elite controllers. Like our data, they also observed a correlation between activation-associated CD28 and CD96+ T cells. Their study linked CD96 downregulation to a terminally differentiated memory phenotype and concluded that the loss of CD96 may contribute to a suboptimal response to HIV infection (77). Consequently, although our study suggests that CD96+ CD8+ T cells in HCV maintain plasticity and memory functions with a higher probability than their CD96- counterparts, further research is needed to explore the bidirectional roles of CD96. Future studies should also include larger, more diverse cohorts, covering other acute and chronic viral and bacterial infections and varying therapeutic regimens, to determine if CD96 regulation is a conserved feature of human immune responses across pathogen-driven disease states. Independent validation of CD96 expression patterns in scRNA-seq and high-dimensional cytometry datasets from HCV and other (chronic) infection cohorts would further boost confidence in the current phenotypic observations. Future research involving functional testing of isolated CD96+ HCV-specific CD8+ T cells will be crucial to determine if the phenotypic differences observed here translate into altered cytotoxicity, proliferative capacity, or recall response. Additional analyses of intrahepatic HCV-specific CD8+ T cells, CD155/CD111 expression on hepatocytes and antigen-presenting cells, and DNAM-1 co-expression on CD96+ versus CD96- HCV-specific CD8+ T cells would help to clarify the ligand-dependent signaling context of CD96 *in vivo*. Additionally, while our phenotypic profiling offers indirect evidence, it is important to directly assess functionality of CD96+ versus CD96– subsets, especially before and after anti-CD96 intervention. Therefore, future studies should compare sorted CD96+ and CD96- CD8+ T cells in various functional assays to see if the phenotypic differences described here result in functional distinctions. Additionally, using antigen-specific CD8+ T cells would allow for consideration of disease-specific effects.

In summary, CD96+ CD8+ T cells were more prevalent in the HCV-specific population compared to the overall CD8+ T-cell pool. Chronic antigen stimulation increased CD96 expression, while transient antigen exposure or HCV eradication decreased it. Phenotypically, the CD96+ subset was enriched for IRF4+, TCF1+, and TOX+ CD8+ T cells, regardless of HCV specificity, may indicating an association between CD96 and a transcriptional or phenotypic program characterized by plasticity and memory-like features rather than terminal exhaustion. This subset contained fewer highly cytolytic T_ec_ cells and higher frequencies of CD39+ CD73- cells, supporting a profile that could be less effector-like and more memory-associated. After HCV-eradication, frequencies of CD96+ PD1+ TIGIT+ HCV-specific CD8+ T cells declined, while their triple-negative counterparts increased, emphasizing the antigen-dependent nature of checkpoint molecule co-expression. Overall, these findings reinforce the notion that CD96 is a distinct checkpoint marker for memory-like HCV-specific CD8+ T cells which may retain greater therapeutic plasticity. This highlights the potential of CD96 as a checkpoint-expressing subset that could be relevant for future therapeutic research. Since we did not directly measure CD96 signaling, CD96 blockade, or combination checkpoint inhibition in CD8+ HCV-specific T cells, the translational implications presented here are speculative and meant to generate hypotheses. It remains to be seen, whether CD96+ HCV-specific CD8+ T cells respond differently to checkpoint blockade or work non-redundantly with anti-PD1 or anti-TIGIT in this disease setting. We therefore frame CD96 as a candidate population distinct from terminally exhausted cells and worth further functional and therapeutic investigation, rather than as an established therapeutic target.

## Data Availability

The raw data supporting the conclusions of this article will be made available by the authors, without undue reservation.

## Supplementary material

Supplementary Figure 1 **(A)** Representative tetramer enrichment with the three resulting fractions: pre (native, before enrichment), depleted and enriched. The cells are gated for the HCV-specific CD8+ T-cell population as tetramer vs. CD8 on total CD3+ T cells. **(B)** Total number of analysed HCV-specific CD8+ T cells of every patient divided into the different disease stages (acute, subacute, chronic, resolved and post-treatment). Created in BioRender. Knapp, M. (2026) https://BioRender.com/09grb0o.

Supplementary Figure 2 Representative gating strategy for identification of HCV-specific CD8+ T cells. First, monocytes, B cells and dead cells were excluded using CD14, CD19 and Live/Dead staining. Then single cells were identified via gating of FSC-A vs. FSC-H. Single cells were gated FSC-A vs. SSC-A for identification of lymphocytes. Detection of T cells was done by gating of CD3 vs. SSC-A. For identification of the CD8+ HCV-specific T cells, tetramer vs. CD8 was gated. Created in BioRender. Knapp, M. (2026) https://BioRender.com/cyeshan.

Supplementary Figure 3 **(A)** Expression of PD1 vs. cell count normalized to mode of all patients in the different stages of HCV-infection with aHCV (green), sHCV (orange), cHCV (black), rHCV (blue) and tHCV (pink). **(B)** MFI of PD1 of HCV-specific CD8+ T cells distinguished between the different stages of HCV infection. **(C)** MFI of PD1 of HCV-specific CD8+ T cells of patients with follow-up samples at first (T_1_, aHCV or sHCV) and second timepoint (T_2_, rHCV or tHCV). Paired values of the patients are shown in same colours with connecting lines. Created in BioRender. Knapp, M. (2026) https://BioRender.com/vffsfgo.

Supplementary Figure 4 **(A)** Expression of TIGIT vs. cell count normalized to mode of all patients in the different stages of HCV-infection with aHCV (green), sHCV (orange), cHCV (black), rHCV (blue) and tHCV (pink). **(B)** MFI of TIGIT of HCV-specific CD8+ T cells distinguished between the different stages of HCV infection. **(C)** MFI of TIGIT of HCV-specific CD8+ T cells of patients with follow-up samples at first (T_1_, aHCV or sHCV) and second timepoint (T_2_, rHCV or tHCV). Paired values of the patients are shown in same colours with connecting lines. Created in BioRender. Knapp, M. (2026) https://BioRender.com/9xncn6c.

Supplementary Figure 5 **(A)** Representative gating for identification of CD39- C73-, CD39- CD73-, CD39- CD73+ and CD39+ CD73+ CD8+ T cells. **(B)** Frequencies of CD39/CD73 subsets gated on bulk (grey) vs. HCV-specific (cyan) CD8+ T cells. **(C)** Frequencies of CD39/CD73 subsets gated on CD96+ bulk (red tiled) and HCV-specific (red) vs. CD96- bulk (blue tiled) and HCV-specific (blue) CD8+ T cells. Created in BioRender. Knapp, M. (2026) https://BioRender.com/o7rzgyq.

Supplementary Figure 6 Frequencies of CD96-PD1-TIGIT subsets gated on bulk (grey) vs. HCV-specific (cyan) CD8+ T cells for all HCV patients and grouped for aHCV, sHCV, cHCV, rHCV and tHCV.

Supplementary Table 1 Detailed demographic, clinical and virologic information from all patients of the study cohort. The table shows patient ID, infection state, sex, age at sample collection, therapy status at sample collection, therapy duration, HLA I, HLA II, HCV genotype, used tetramer epitopes, sequenced epitopes of the patient samples, IL-28 allele, viral load, bilirubin, aspartate aminotransferase (AST), alanine aminotransferase (ALT), gamma-glutamyl transferase (GGT), alkaline phosphatase (AP), C-reactive protein (CRP) and international normalized ratio (INR). All laboratory parameters are measured at date of sample collection. The HLA I types used for our tetramer staining are marked in bold. Mutations in the viral sequences which deviate from the used epitope are marked in bold.

Supplementary Table 2 Flow cytometry panel. Overview of used antibodies with corresponding lasers and filters of the BD FACSymphony™ A3 Cell Analyzer.

Supplementary Table 3 Total number of analysed HCV-specific CD8+ T cells of every patient divided into the different disease stages (acute, subacute, chronic, resolved and post-treatment) and total number of analysed HCV-specific CD8+ T cells per group.
