# Supplementary material for: CD96 marks a phenotypically distinct checkpoint-associated HCV-specific CD8+ T-cell subset featuring memory-associated states
Source: Front Immunol. 2026 Jul 13;17:1883007. doi: 10.3389/fimmu.2026.1883007 (PMC13402549; doi:10.3389/fimmu.2026.1883007)
Supplement: Supplementary Figure 1 — (A) Representative tetramer enrichment with the three resulting fractions: pre (native, before enrichment), depleted and enriched. The cells are gated for the HCV-specific CD8+ T-cell population as tetramer vs. CD8 on total CD3+ T cells. (B) Total number of analysed HCV-specific CD8+ T cells of every patient divided into the different disease stages (acute, subacute, chronic, resolved and post-treatment). Created in BioRender. Knapp, M. (2026) https://BioRender.com/09grb0o. [file DataSheet1.zip › Suppl. Table 3.docx]

| Project ID | Group | # of Tet+ T cells | # of Tet+ T cells per group |
| --- | --- | --- | --- |
| HCV01 | aHCV | 6,111 | 35,789 |
| HCV28 |  | 110 |  |
| HCV14 |  | 102 |  |
| HCV19 |  | 5,105 |  |
| HCV22 |  | 50 |  |
| HCV10 |  | 336 |  |
| HCV32 |  | 23,975 |  |
| HCV06 | sHCV | 37 | 1,500 |
| HCV17 |  | 934 |  |
| HCV12 |  | 262 |  |
| HCV07 |  | 267 |  |
| HCV24 | cHCV | 20 | 1,815 |
| HCV25 |  | 334 |  |
| HCV08 |  | 70 |  |
| HCV04 |  | 103 |  |
| HCV26 |  | 95 |  |
| HCV09 |  | 134 |  |
| HCV29 |  | 91 |  |
| HCV31 |  | 155 |  |
| HCV27 |  | 163 |  |
| HCV30 |  | 220 |  |
| HCV18 |  | 326 |  |
| HCV05 |  | 104 |  |
| HCV19 | rHCV | 51 | 2,437 |
| HCV02 |  | 22 |  |
| HCV10 |  | 270 |  |
| HCV21 |  | 109 |  |
| HCV11 |  | 254 |  |
| HCV13 |  | 96 |  |
| HCV15 |  | 1,635 |  |
| HCV20 | tHCV | 1,144 | 13,426 |
| HCV22 |  | 54 |  |
| HCV16 |  | 11,030 |  |
| HCV06 |  | 384 |  |
| HCV12 |  | 60 |  |
| HCV14 |  | 147 |  |
| HCV03 |  | 86 |  |
| HCV23 |  | 521 |  |
| in total: |  |  | 54,967 |
