# Supplementary figures and images for: CD96 marks a phenotypically distinct checkpoint-associated HCV-specific CD8+ T-cell subset featuring memory-associated states
Source: Front Immunol. 2026 Jul 13;17:1883007. doi: 10.3389/fimmu.2026.1883007 (PMC13402549; doi:10.3389/fimmu.2026.1883007)

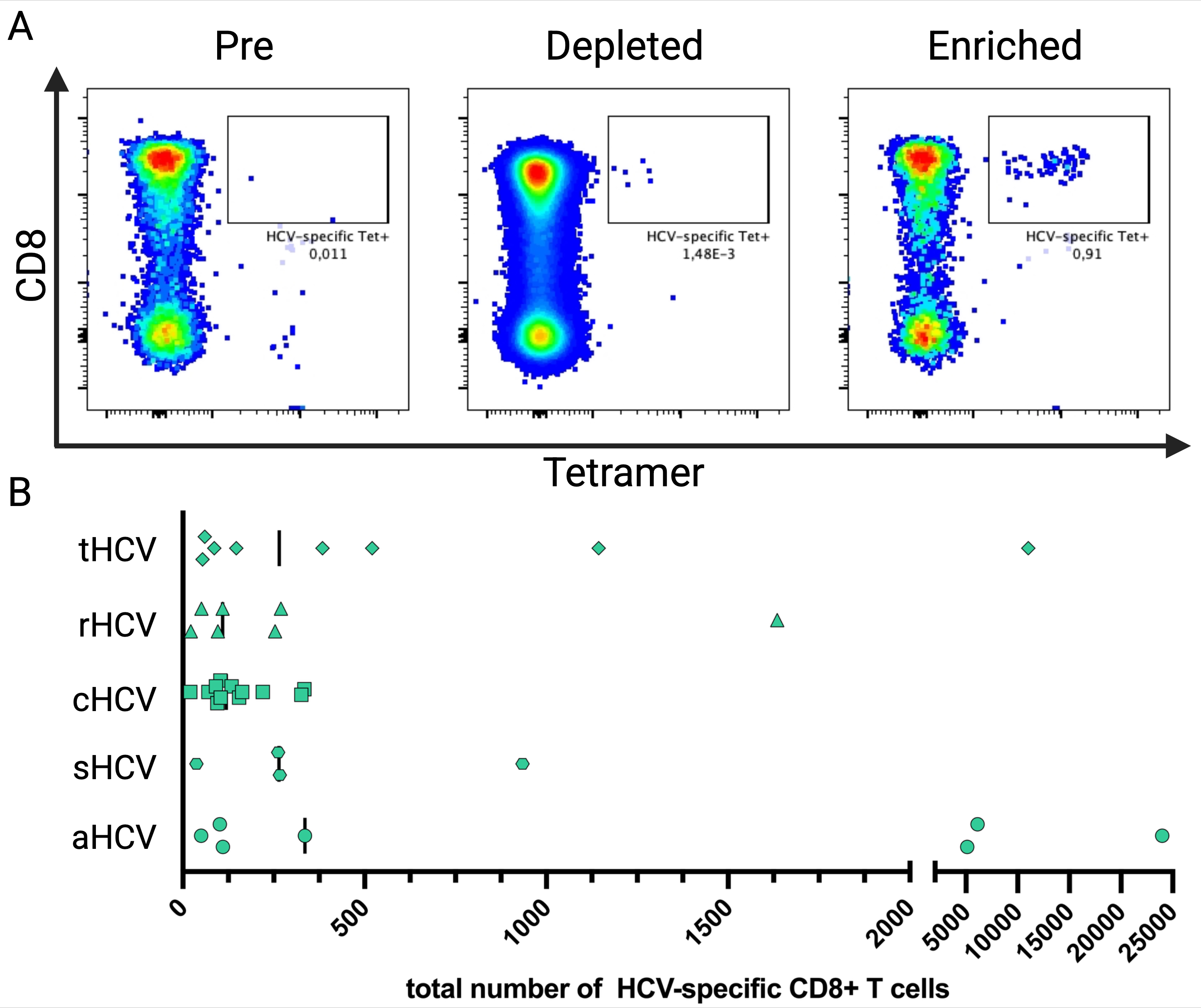

Supplement: Supplementary Figure 1 — (A) Representative tetramer enrichment with the three resulting fractions: pre (native, before enrichment), depleted and enriched. The cells are gated for the HCV-specific CD8+ T-cell population as tetramer vs. CD8 on total CD3+ T cells. (B) Total number of analysed HCV-specific CD8+ T cells of every patient divided into the different disease stages (acute, subacute, chronic, resolved and post-treatment). Created in BioRender. Knapp, M. (2026) https://BioRender.com/09grb0o. [file DataSheet1.zip › Suppl. Fig. 1.jpeg]

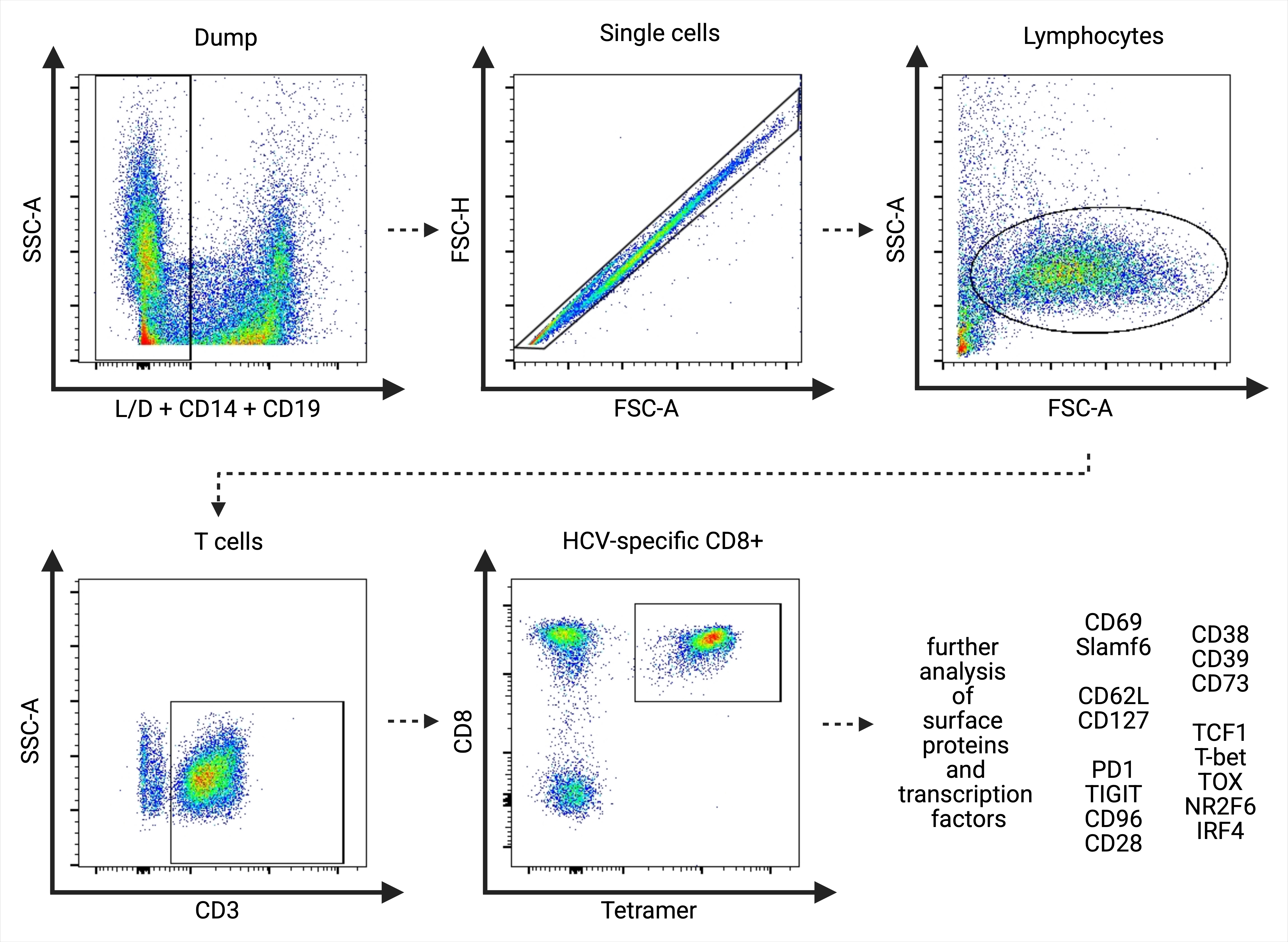

Supplement: Supplementary Figure 1 — (A) Representative tetramer enrichment with the three resulting fractions: pre (native, before enrichment), depleted and enriched. The cells are gated for the HCV-specific CD8+ T-cell population as tetramer vs. CD8 on total CD3+ T cells. (B) Total number of analysed HCV-specific CD8+ T cells of every patient divided into the different disease stages (acute, subacute, chronic, resolved and post-treatment). Created in BioRender. Knapp, M. (2026) https://BioRender.com/09grb0o. [file DataSheet1.zip › Suppl. Fig. 2.jpeg]

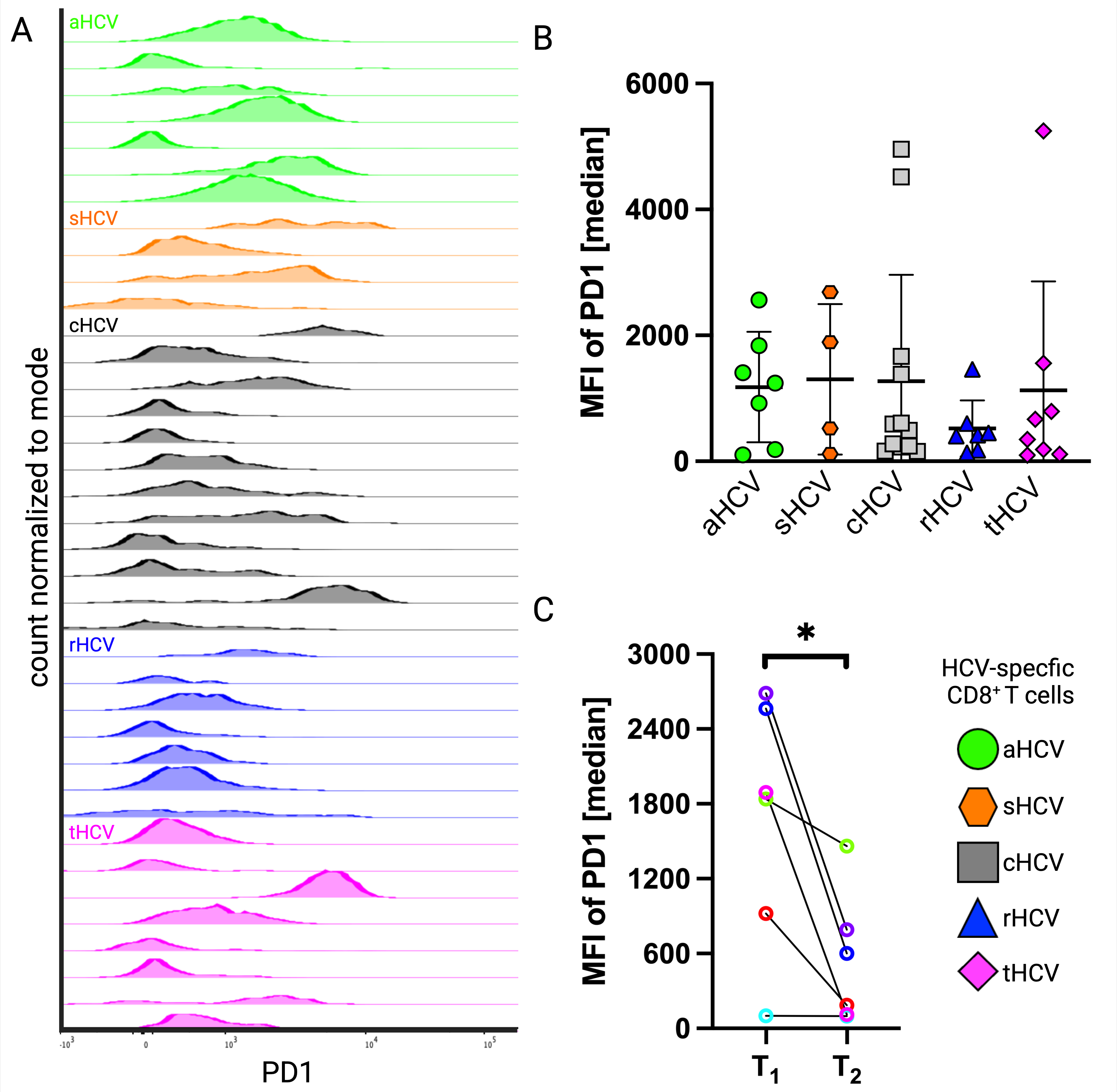

Supplement: Supplementary Figure 1 — (A) Representative tetramer enrichment with the three resulting fractions: pre (native, before enrichment), depleted and enriched. The cells are gated for the HCV-specific CD8+ T-cell population as tetramer vs. CD8 on total CD3+ T cells. (B) Total number of analysed HCV-specific CD8+ T cells of every patient divided into the different disease stages (acute, subacute, chronic, resolved and post-treatment). Created in BioRender. Knapp, M. (2026) https://BioRender.com/09grb0o. [file DataSheet1.zip › Suppl. Figure 3.jpeg]

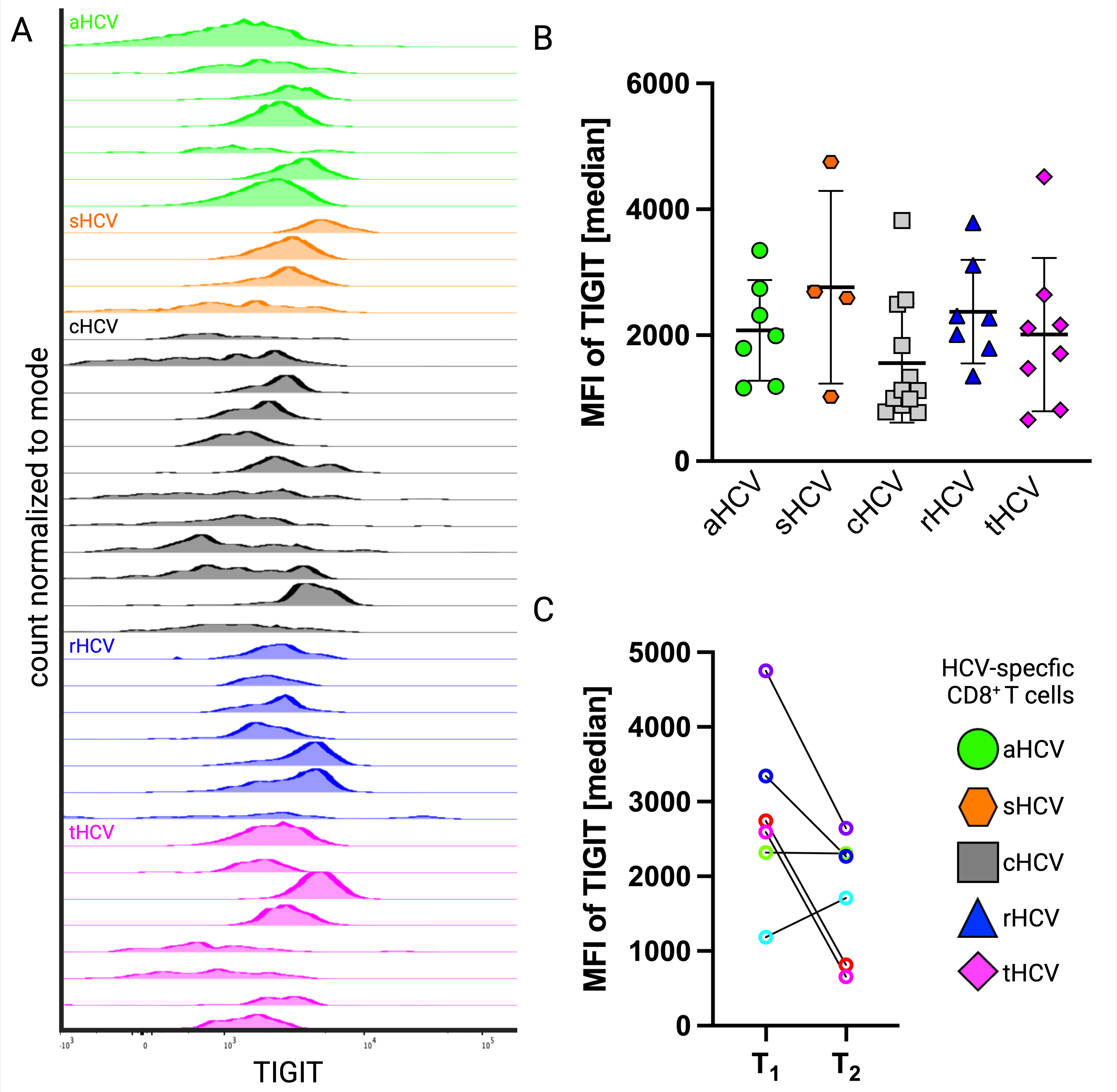

Supplement: Supplementary Figure 1 — (A) Representative tetramer enrichment with the three resulting fractions: pre (native, before enrichment), depleted and enriched. The cells are gated for the HCV-specific CD8+ T-cell population as tetramer vs. CD8 on total CD3+ T cells. (B) Total number of analysed HCV-specific CD8+ T cells of every patient divided into the different disease stages (acute, subacute, chronic, resolved and post-treatment). Created in BioRender. Knapp, M. (2026) https://BioRender.com/09grb0o. [file DataSheet1.zip › Suppl. Figure 4.jpeg]

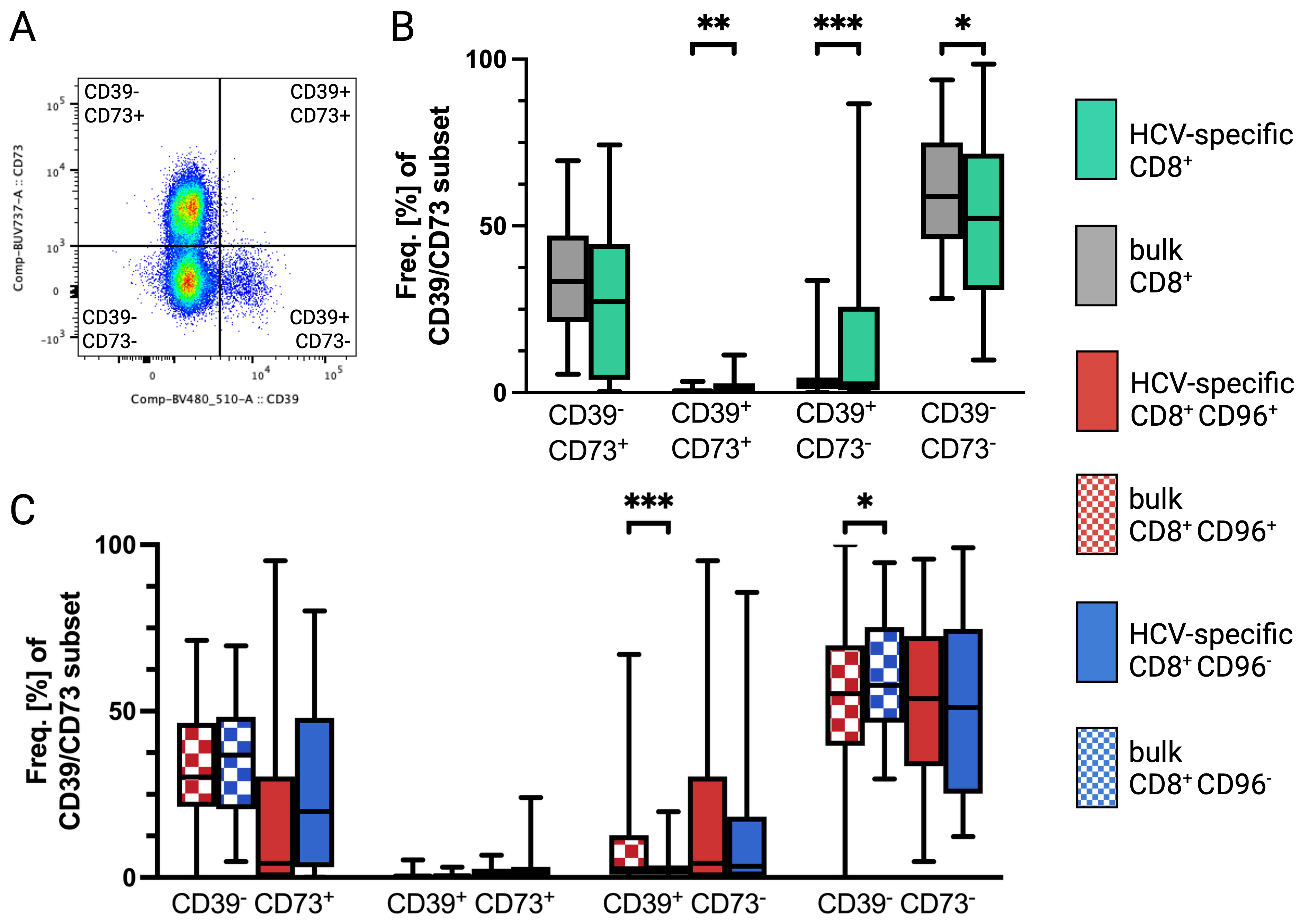

Supplement: Supplementary Figure 1 — (A) Representative tetramer enrichment with the three resulting fractions: pre (native, before enrichment), depleted and enriched. The cells are gated for the HCV-specific CD8+ T-cell population as tetramer vs. CD8 on total CD3+ T cells. (B) Total number of analysed HCV-specific CD8+ T cells of every patient divided into the different disease stages (acute, subacute, chronic, resolved and post-treatment). Created in BioRender. Knapp, M. (2026) https://BioRender.com/09grb0o. [file DataSheet1.zip › Suppl. Figure 5.jpeg]

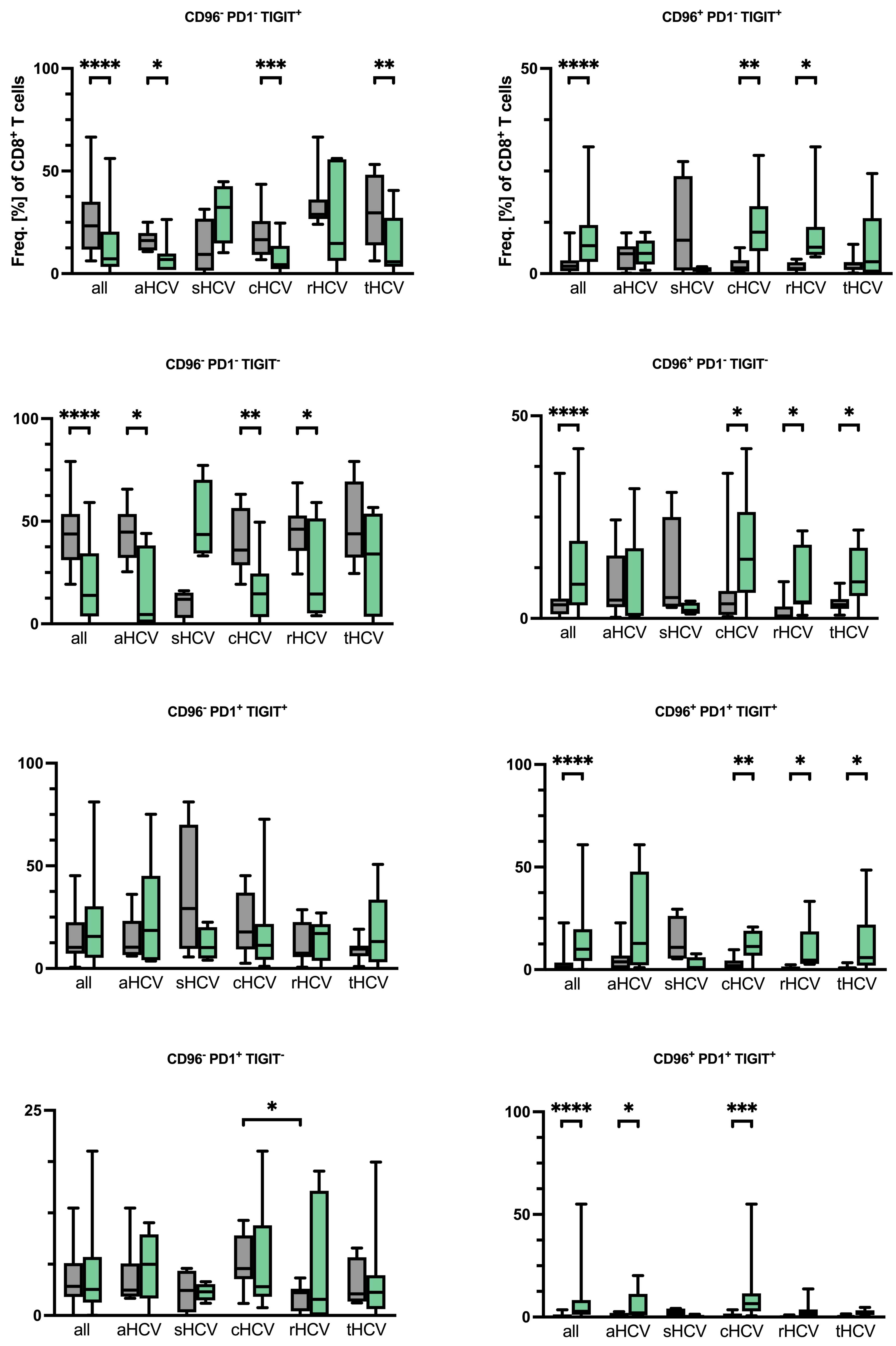

Supplement: Supplementary Figure 1 — (A) Representative tetramer enrichment with the three resulting fractions: pre (native, before enrichment), depleted and enriched. The cells are gated for the HCV-specific CD8+ T-cell population as tetramer vs. CD8 on total CD3+ T cells. (B) Total number of analysed HCV-specific CD8+ T cells of every patient divided into the different disease stages (acute, subacute, chronic, resolved and post-treatment). Created in BioRender. Knapp, M. (2026) https://BioRender.com/09grb0o. [file DataSheet1.zip › Suppl. Figure 6.jpeg]

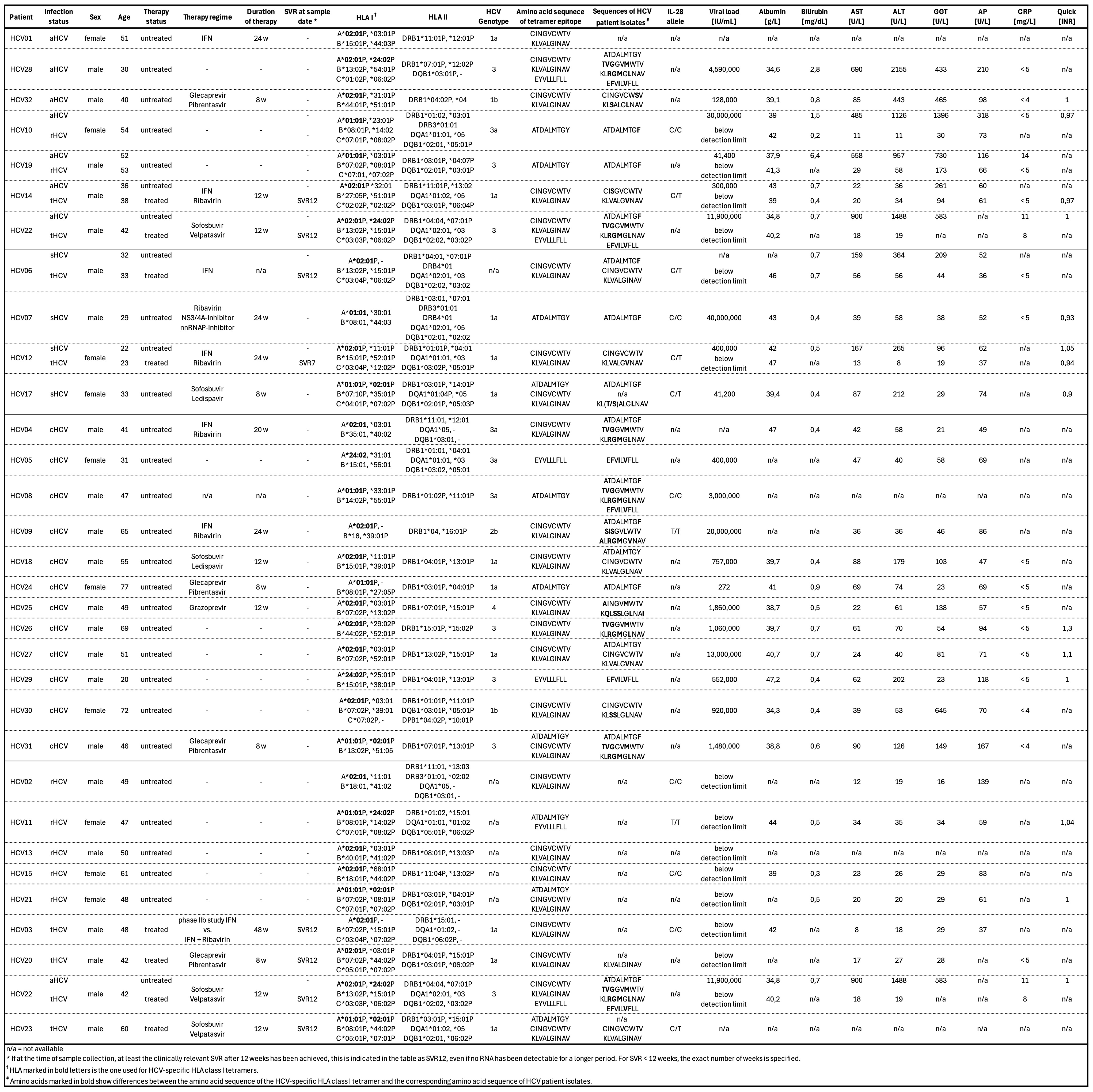

Supplement: Supplementary Figure 1 — (A) Representative tetramer enrichment with the three resulting fractions: pre (native, before enrichment), depleted and enriched. The cells are gated for the HCV-specific CD8+ T-cell population as tetramer vs. CD8 on total CD3+ T cells. (B) Total number of analysed HCV-specific CD8+ T cells of every patient divided into the different disease stages (acute, subacute, chronic, resolved and post-treatment). Created in BioRender. Knapp, M. (2026) https://BioRender.com/09grb0o. [file DataSheet1.zip › Suppl. Table 1.jpeg]

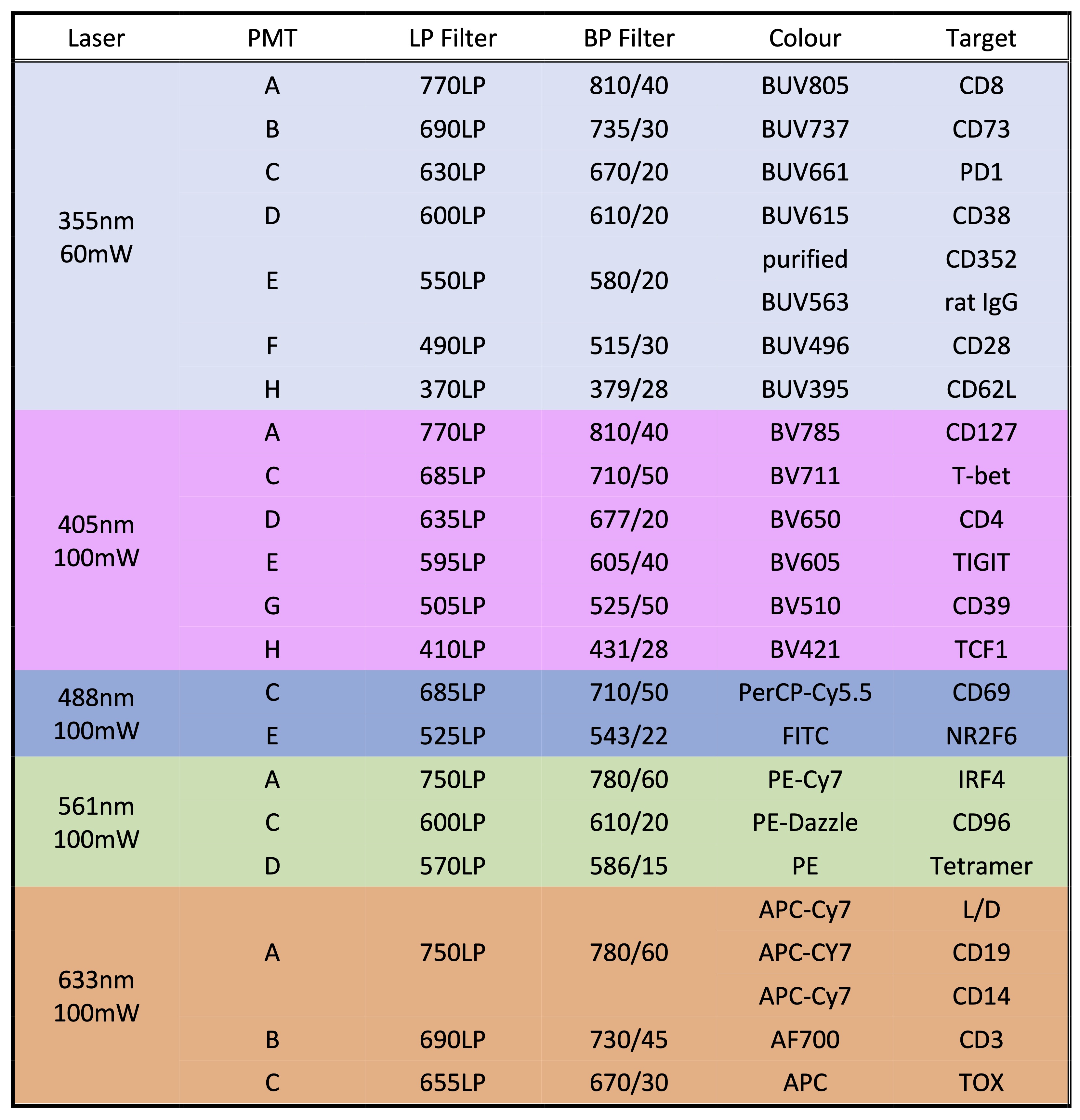

Supplement: Supplementary Figure 1 — (A) Representative tetramer enrichment with the three resulting fractions: pre (native, before enrichment), depleted and enriched. The cells are gated for the HCV-specific CD8+ T-cell population as tetramer vs. CD8 on total CD3+ T cells. (B) Total number of analysed HCV-specific CD8+ T cells of every patient divided into the different disease stages (acute, subacute, chronic, resolved and post-treatment). Created in BioRender. Knapp, M. (2026) https://BioRender.com/09grb0o. [file DataSheet1.zip › Suppl. Table 2.jpeg]
